# Molecularly Imprinted Polymers Based on Cyclodextrin Derivatives and Chitosan for Selective Extraction of Drugs and Dyes

**DOI:** 10.3390/ijms27167502

**Published:** 2026-08-21

**Authors:** Linara Kopnova, Alexander Kopnov, Igor Zlotnikov, Elena Kudryashova

**Affiliations:** 1 Autonomous Non-Profit Higher Education Organization “Central University”, 125212 Moscow, Russia; yakupova.linara@mail.ru (L.K.);; 2 Chemistry Department, Lomonosov Moscow State University, 119991 Moscow, Russia

**Keywords:** molecularly imprinted polymers, β-cyclodextrin, chitosan, genipin, selective sorption, levofloxacin, fluorescein, analysis of biological and food samples

## Abstract

A series of molecularly imprinted polymers (MIPs) based on hydroxypropyl-β-cyclodextrin (HPCD) crosslinked with 1,6-hexamethylene diisocyanate (HMD) or toluene diisocyanate (TDI), as well as hybrid chitosan–HPCD polymers crosslinked with genipin, were synthesized using levofloxacin and fluorescein as template molecules. The structure and spatial organization of the obtained materials were characterized by FTIR spectroscopy, FTIR microscopy mapping, and ζ-potential measurements. The influence of pH, crosslinker content, and template structure on sorption performance was investigated. All MIPs exhibited maximum sorption at pH 3.0. The highest sorption capacity toward levofloxacin was achieved for the LV–Chit–HPCD–Genipin_MIP_ (74.8 mg/g), whereas the fluorescein-imprinted FL–HPCD–TDI_MIP_ (1:1) demonstrated the highest sorption capacity (135.5 mg/g) and selectivity coefficient (84.1). Dynamic column experiments confirmed efficient analyte extraction, reducing the analyte concentration by more than 90% after ten loading cycles. All synthesized MIPs exhibited excellent regenerability, with less than 3% loss of sorption efficiency after ten consecutive sorption–desorption cycles. The applicability of the developed sorbents to real matrices was demonstrated using milk and blood plasma samples after minimal sample preparation. Fluorescein extraction efficiencies reached 97.6% and 93.6% for milk and plasma, respectively. The obtained results demonstrate that HPCD-based MIPs combine high sorption capacity, exceptional selectivity, operational stability, and applicability to complex biological matrices, making them promising materials for selective sample preparation, analyte preconcentration, and controlled drug delivery systems.

## 1. Introduction

Current challenges in biomedical analysis and environmental monitoring require the development of sorbents capable of selectively binding target compounds (analytes) in the presence of structurally related species within complex multicomponent mixtures. Conventional sorbents, such as silica gels and ion-exchange resins, despite their high overall sorption capacity, often lack sufficient selectivity, thereby limiting the efficiency of separation and concentration processes. In this context, polymeric systems exhibiting selective recognition toward target analytes have attracted considerable interest. Molecularly imprinted polymers (MIPs) are synthetic materials in which recognition sites complementary to the template molecule in terms of the spatial arrangement of functional groups are formed during polymerization. These specific binding sites provide high selectivity toward the target analyte (template molecule), allowing MIPs to be regarded as synthetic analogs of biological antibodies without their inherent drawbacks. Environmental and biomedical applications represent the most actively developing fields for MIP implementation. The ability of these polymers to selectively bind target analytes in complex matrices, including natural waters and biological fluids, enables the detection of pollutants and pharmaceutical compounds at trace concentrations without laborious sample preparation [1,2,3,4,5,6,7,8]. This creates opportunities for the development of portable sensing systems and efficient approaches for monitoring and/or removing pharmaceutical residues, dyes, and other contaminants from wastewater, including fluoroquinolone (levofloxacin and ciprofloxacin), carbapenems (meropenem) and other antibiotics, which are routinely detected in treated wastewater effluents at total concentrations of 1730–2840 ng/L and are associated with the proliferation of antibiotic-resistant bacteria [9]; synthetic xanthene dyes such as rhodamine 6G and fluorescein, which enter aquatic environments through textile industry effluents and exhibit confirmed carcinogenic and mutagenic properties [10]; and endocrine-disrupting compounds such as bisphenol A, detected in industrial and domestic wastewater at concentrations of 16–1465 ng/L and in surface waters at 170–3113 ng/L [11].

For example, the authors of [12] reported the development of the MIP for the selective preconcentration of albendazole followed by HPLC-UV detection. The resulting material exhibited a sorption capacity of 34.9 mg/g and a detection limit of 1.46 ng/mL and enabled analyte determination in biological fluids with high selectivity and reproducibility. In another study [13], an electrochemical sensor based on an MIP modified with carbon nanotubes and gold nanoparticles was developed for bisphenol A determination. The sensor achieved a detection limit of 5 nM and a linear range of 0.01–10 μM under optimized conditions and was successfully applied to the analysis of real milk samples. Furthermore, the authors of [14] reported the synthesis of a magnetic porous MIP for the selective extraction of tetracycline antibiotics. The obtained materials exhibited sorption capacities of up to 14.5 mg/g and selectivity coefficients as high as 36.9.

The application of MIPs to the analysis of complex biological and food matrices, particularly milk and blood plasma, is of considerable practical importance, since monitoring pharmaceutical compounds and toxicants in these media is essential for food safety assurance, therapeutic drug monitoring, and pharmacokinetic studies. Conventional analytical approaches based mainly on HPLC-MS/MS, gas chromatography, and enzyme-linked immunosorbent assays require multistep sample preparation procedures, expensive instrumentation, and/or biological reagents with limited shelf life. Therefore, selective concentration using MIPs is considered a promising alternative. Thus, the authors of [4] reported the preparation of MIPs for the solid-phase extraction of fluoroquinolones from milk followed by chromatographic detection. The obtained sorbent provided recoveries ranging from 76.8 to 97.7% and detection limits of 10–20 ng/mL. In another study [15], magnetic surface molecularly imprinted polymers were developed for the determination of roxithromycin in human plasma by HPLC-MS/MS. The method exhibited a detection limit of 9.8 ng/mL and recoveries of 86.5–91.5%, demonstrating excellent extraction efficiency and reproducibility. Moreover, ref. [16] described a molecularly imprinted sorbent for the determination of naftopidil in plasma and urine, providing efficient analyte preconcentration prior to chromatographic analysis. Detection limits of 7.5 and 4.0 ng/mL were achieved for plasma and urine samples, respectively, with recoveries of 90–100%. These studies highlight the high potential of MIPs as materials for sample preparation and trace-level determination of analytes in various matrices. Nevertheless, despite significant advances in the development of MIPs for complex biological and food matrices, many published studies have focused primarily on improving sorption capacity and analytical sensitivity, whereas the selectivity and ability of polymers to discriminate between structurally related compounds remain insufficiently investigated. However, high selectivity is a critical prerequisite for the analytical application of MIPs in multicomponent systems such as milk, blood serum, and other biological fluids. Therefore, further development of MIPs aimed at enhancing their selectivity and binding affinity represents a relevant and promising research direction.

β-Cyclodextrin derivatives are considered promising monomers for the preparation of MIPs owing to their unique structure combining a hydrophobic internal cavity with a hydrophilic external surface. This architecture enables β-cyclodextrin derivatives to form various non-covalent complexes (in particular, the “guest–host” mechanism and the mechanism of external association) with a broad range of molecules. In addition, β-cyclodextrin derivatives possess reactive functional groups (hydroxyl, amino, sulfobutyl, etc.) located on the macrocyclic periphery, allowing chemical modification and the fabrication of polymeric structures based on these molecules [17,18,19,20]. In the present work, hydroxypropyl-β-cyclodextrin (HPCD) was employed as a monomer because of its high aqueous solubility (>600 mg/mL) and its ability to react with various bifunctional crosslinking agents.

An important factor determining the sorption properties of polymeric materials is the nature and stoichiometry of the crosslinker. Variation in the linker structure makes it possible to regulate matrix rigidity, accessibility of imprinted sites, and affinity toward target analytes, thereby directly affecting sorption capacity and selectivity. In this work, toluene diisocyanate (TDI) and 1,6-hexamethylene diisocyanate (HMD) were selected as crosslinkers because the flexible hydrophobic hexamethylene fragment of HMD promotes the formation of hydrophobic pores, increasing affinity toward nonpolar molecules, whereas the aromatic moiety of TDI provides matrix rigidity and additional interactions with aromatic fragments of template molecules.

The development of hybrid materials represents another promising direction in sorbent design. Chitosan-based composites combine the advantages of molecular imprinting with the biocompatibility and biodegradability of this natural polysaccharide [21,22,23]. The presence of free amino groups in chitosan facilitates additional electrostatic interactions with negatively charged analytes. Combining chitosan with cyclodextrin enables the formation of a three-dimensional architecture integrating the advantages of both components. To introduce crosslinks in chitosan–HPCD hybrid polymers, genipin, a natural compound isolated from the fruits of *Gardenia jasminoides Ellis*, was selected as a crosslinking agent. Compared to conventionally used glutaraldehyde, genipin exhibits substantially lower cytotoxicity and superior biocompatibility, making it particularly attractive for the development of biomaterials. The reaction between genipin and amino groups of chitosan results in the formation of a chemically and mechanically stable three-dimensional network characterized by enhanced resistance to hydrolysis and the ability to preserve structural integrity under physiological conditions [24,25].

The combination of molecular imprinting with cyclodextrin-based host–guest interactions and biocompatible polymer matrices offer a promising strategy for the development of highly selective sorbents capable of recognizing structurally related compounds in complex multicomponent systems. Such materials are of particular interest for sample preparation, selective preconcentration, and trace analysis of pharmaceuticals and organic contaminants in environmental, food, and biological samples. Therefore, the development of novel cyclodextrin-based MIPs with enhanced affinity, selectivity, and applicability to real matrices such as milk and blood plasma represents an important and timely research direction.

## 2. Results

### 2.1. Synthesis of MIPs

**Figure 1:**
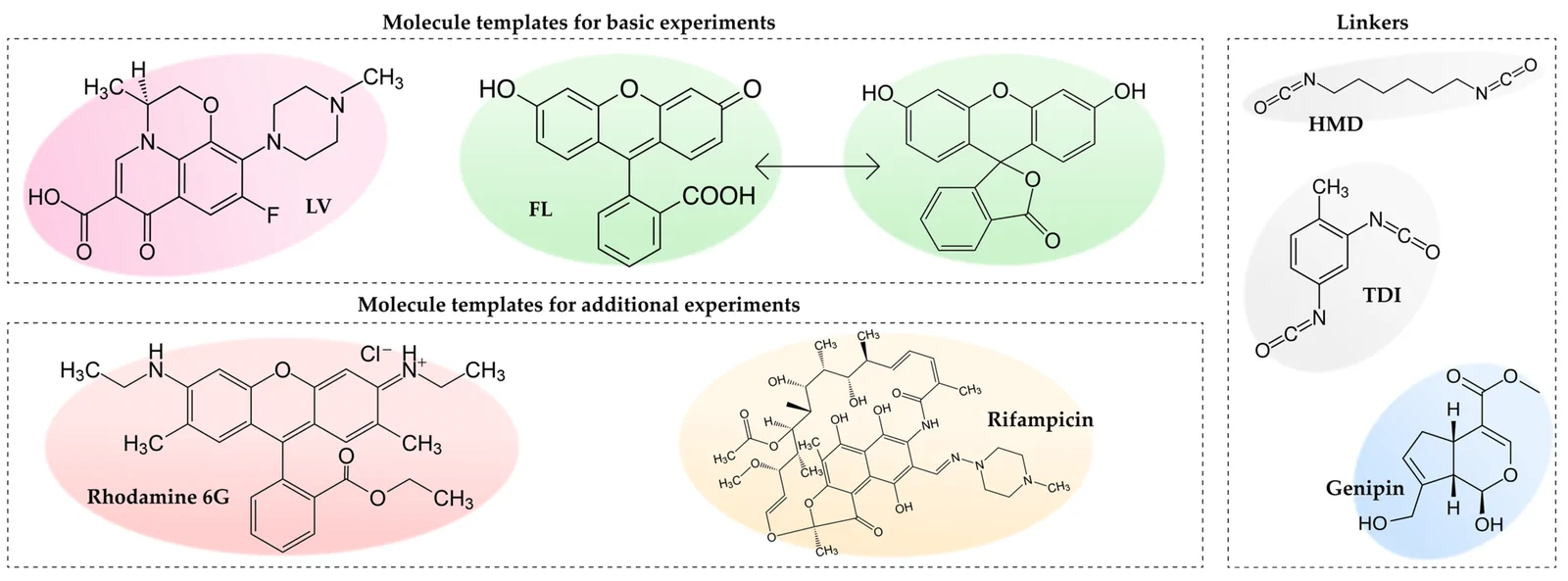
Structural formulas of template molecules (LV, FL, rhodamine 6G, rifampicin) and crosslinking agents (TDI, HMD and genipin). For fluorescein, two structural forms are presented: the closed lactone form, which predominates under acidic conditions, and the open quinoid form, which is characteristic of neutral and alkaline conditions.

In the present work, a series of MIPs based on hydroxypropyl-β-cyclodextrin and chitosan were developed as promising selective sorbents for the recognition, extraction, and preconcentration of environmentally and biologically relevant compounds. Owing to the combination of cyclodextrin-based host–guest interactions and molecular imprinting, these materials are expected to exhibit high affinity and selectivity toward structurally defined target molecules. To evaluate the sorption performance and recognition properties of the synthesized polymers, pharmaceutical compounds and organic dyes were selected as model analytes. Specifically, the antibacterial agents, levofloxacin (LV) and rifampicin, as well as the dyes fluorescein (FL) and rhodamine 6G, were employed in this study (Figure 1). Particular emphasis was placed on levofloxacin and fluorescein because their molecular structures possess common features, including comparable molecular size, aromatic fragments, and the presence of carboxyl and ether groups. Therefore, comparison of the sorption behavior of the polymers toward these two compounds provides a convenient model for assessing the selectivity of the obtained MIPs.

**Figure 2:**
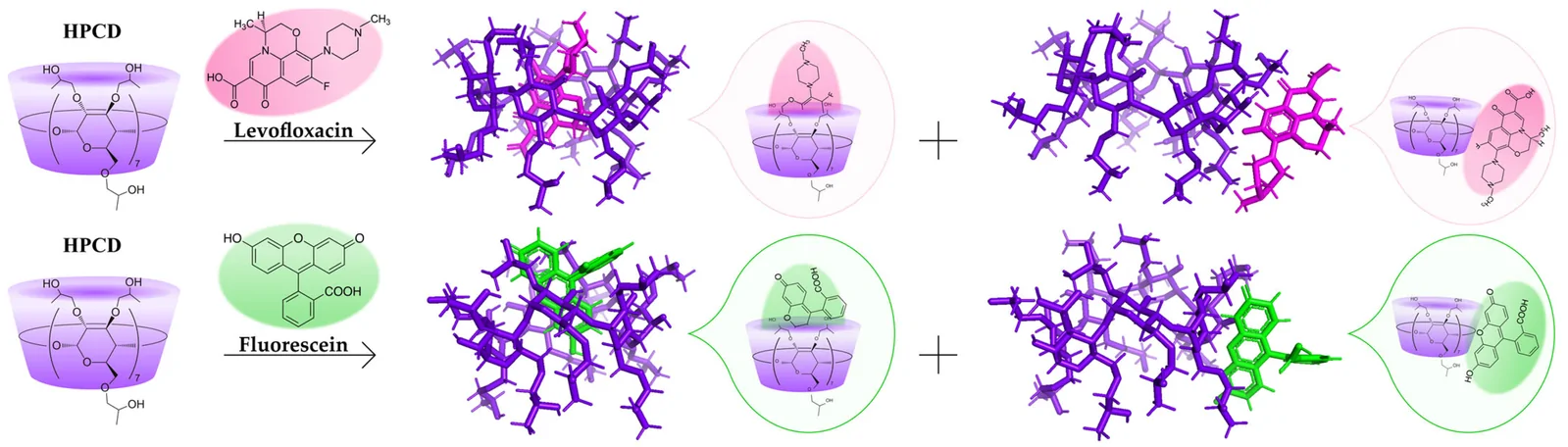
The scheme of formation of the HPCD complex with levofloxacin/fluorescein and 3D visualization of the resulting complexes.

As discussed above, cyclodextrin derivatives are capable of forming host–guest inclusion complexes with a wide variety of organic molecules due to the presence of a hydrophobic internal cavity and a hydrophilic outer surface. Therefore, prior to polymer synthesis, the possible modes of interaction between HPCD and the selected template molecules were analyzed based on published data and molecular modeling results (Figure 2). The internal cavity diameter of β-cyclodextrin (approximately 6.0–6.5 Å [26]) is well-suited for accommodating aromatic and hydrophobic fragments of both levofloxacin and fluorescein, whose characteristic molecular dimensions are comparable to the cyclodextrin cavity size. The literature data indicate that fluoroquinolones readily form inclusion complexes with cyclodextrins through penetration of the quinolone aromatic fragment into the cavity, while additional stabilization is provided by hydrogen bonding and external association mechanisms [27,28]. Although direct evidence for the formation of HPCD–fluorescein inclusion complexes remains limited, the literature reports describing the interaction of cyclodextrins with dyes [29] indicate that aromatic chromophores of suitable size can participate in host–guest complexation. Based on these observations and the structural features of fluorescein, a similar mode of interaction involving partial inclusion of the xanthene fragment into the cyclodextrin cavity appears plausible. The experimentally determined dissociation constants were 9.6 × 10^−4^ M for the HPCD–LV complex and 7.5 × 10^−4^ M for the HPCD–FL complex, indicating the formation of moderately stable supramolecular assemblies typical of cyclodextrin-based host–guest systems. The lower *K_dis_* value obtained for fluorescein suggests a somewhat higher affinity toward HPCD compared to levofloxacin, which may be attributed to the extended aromatic xanthene fragment capable of establishing stronger hydrophobic interactions with the cyclodextrin cavity. In addition to classical inclusion complexation, external association involving hydroxyl groups located on the cyclodextrin rim is also likely to contribute to complex stabilization. Such cooperative interactions are particularly important for molecular imprinting, as they facilitate the formation and stabilization of recognition sites during polymerization. The proposed HPCD–template structures were therefore considered based not only on the structural features of the interacting molecules but also on previously obtained by our group data [30]. For the LF–HPCD system, previous ATR-FTIR and molecular dynamics studies demonstrated a 1:1 complex stoichiometry and a dissociation constant of approximately 1.0 × 10^−3^ M. Molecular modeling showed deep inclusion of the quinolone aromatic core into the CD cavity and the immersion of the carboxyl group, together with hydrophobic contacts and hydrogen-bonding interactions involving the carboxyl and piperazine groups. The calculated complex-formation energies for the studied fluoroquinolone–CD systems were negative, ranging from −16 to −35 kcal/mol, and showed good agreement with the experimentally determined values, confirming the energetically favorable nature of complex formation. Based on these experimentally supported structural and energetic characteristics, and using the previously established interaction patterns as a reference, the proposed structures of the HPCD–template complexes with levofloxacin and fluorescein were visualized using PyMOL Molecular Graphics System (version 3.1.8, Schrödinger, LLC, New York, NY, USA).

**Figure 3:**
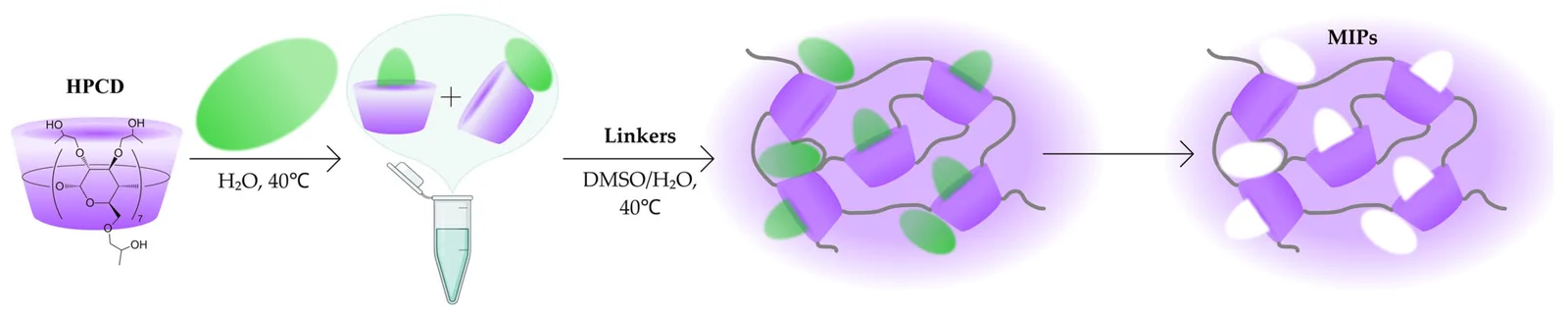
The scheme of synthesis of MIPs based on HPCD.

MIPs based on HPCD were synthesized using HMD and TDI as crosslinking agents. The preparation of the sorbents was carried out in several sequential steps (Figure 3). In the first step, a non-covalent inclusion complex between HPCD and the template molecule was formed; in the second step, the complex was polymerized in the presence of a diisocyanate crosslinker, yielding a covalently crosslinked three-dimensional polymer network. As a control, non-imprinted polymers (NIP) without template molecules were synthesized. In the final step, the obtained MIPs were purified from unreacted reagents and residual template molecules by equilibrium dialysis. This procedure ensured the liberation of the imprinted cavities, rendering them available for selective analyte binding. Complete removal of the template was confirmed by the absence of characteristic absorption bands in the UV spectra of the dialysate fractions.

**Figure 4:**
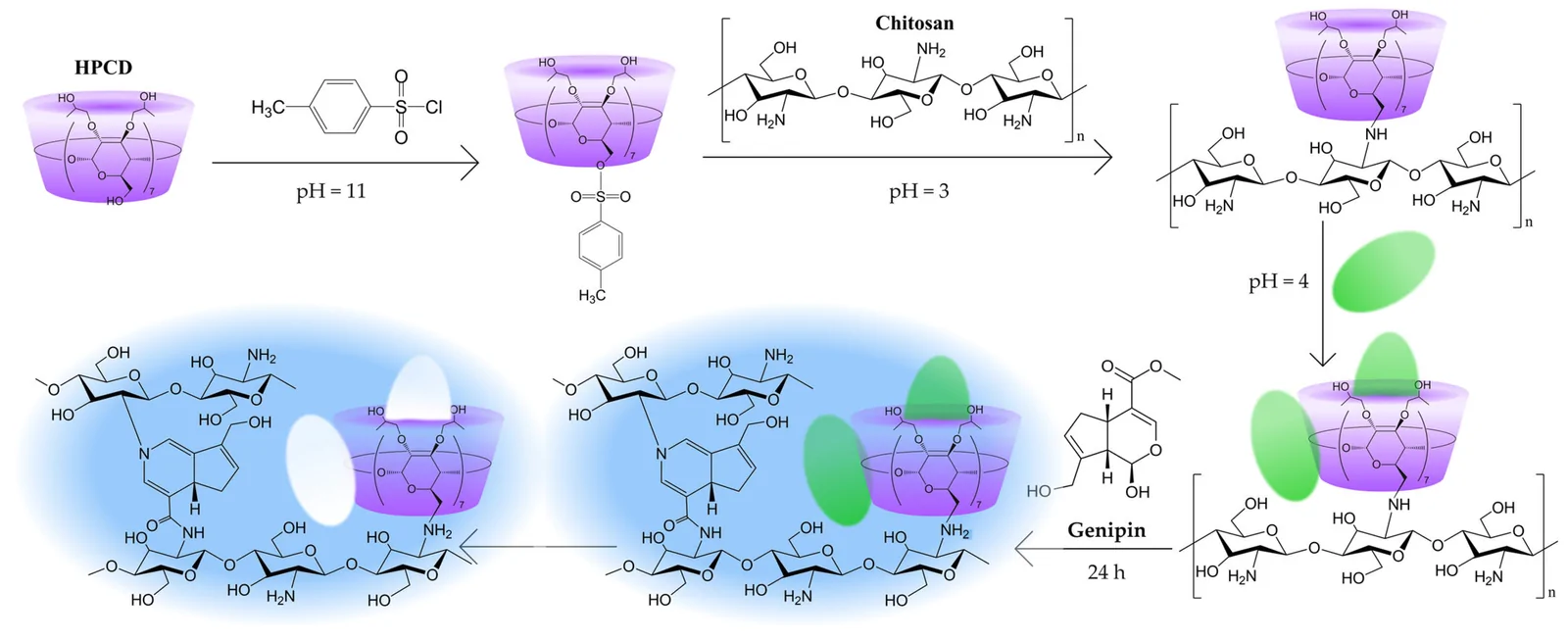
The scheme of synthesis of MIPs based on chitosan and HPCD.

The synthesis of composite MIPs based on chitosan and HPCD was performed in several consecutive steps (Figure 4). In the preliminary step, HPCD was tosylated: introduction of a tosyl group at a hydroxyl position of the macrocycle generated an effective leaving group required for subsequent covalent attachment to chitosan. In the following step, tosyl–HPCD was reacted with the amino groups of chitosan; nucleophilic substitution at the nitrogen atom established a covalent C–N linkage, ensuring stable incorporation of the cyclodextrin moiety into the polymer backbone. After purification of the resulting chitosan–HPCD conjugate, it was incubated with levofloxacin to allow the formation of a non-covalent host–guest complex. In the final step, crosslinking of the system was carried out using genipin, which forms covalent bonds with the amino groups of chitosan not engaged in HPCD attachment, thereby generating a three-dimensional polymer network that stabilizes the imprinted cavities and preserves their geometric complementarity to the template molecule. Removal of the template from the synthesized MIP was accomplished by equilibrium dialysis.

### 2.2. Investigation of the Structure of Synthesized Polymers

#### 2.2.1. FTIR Spectroscopy

**Figure 5:**
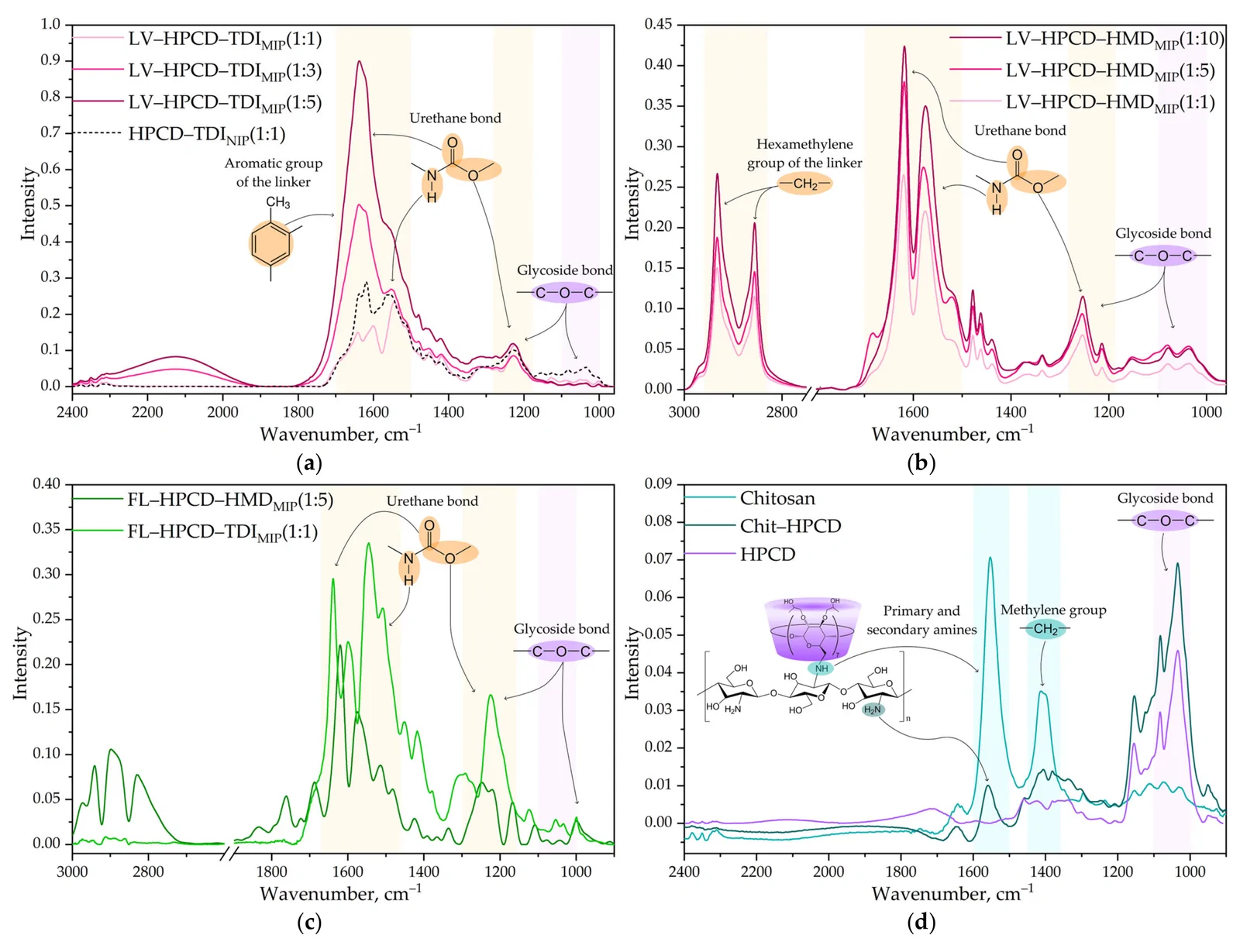

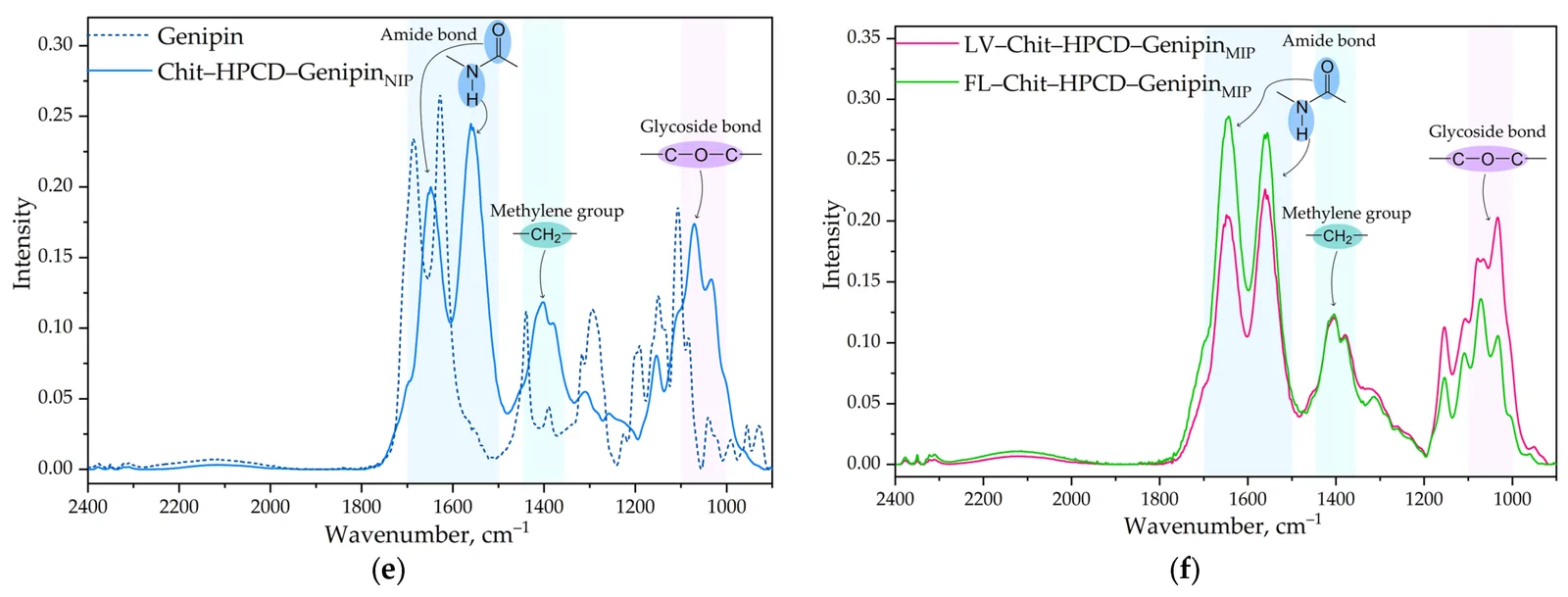
FTIR spectra of: (**a**) levofloxacin’s MIPs based on HPCD crosslinked with TDI in various molar excesses of the linker, and non-imprinted polymer HPCD–TDI_NIP_ (1:1) without a template molecule; (**b**) of levofloxacin’s MIPs based on HPCD crosslinked with HMD in various molar excesses of the linker; (**c**) fluorescein’s MIPs based on HPCD crosslinked with TDI and HMD in molar excesses of the linker 1:1 and 1:5, respectively; (**d**) chitosan (Chit), HPCD and Chit–HPCD conjugate; (**e**) genipin (G) and non-imprinted polymer Chit– HPCD–Genipin_NIP_ without a template molecule; (**f**) levofloxacin’s and fluorescein’s MIPs based on Chit–HPCD conjugate crosslinked with genipin.

To confirm the structure of the synthesized MIPs after template removal, Fourier-transform infrared (FTIR) spectroscopy was employed (Figure 5). The spectra of all HPCD-based MIPs exhibited characteristic absorption bands of HPCD in the 1000–1100 cm^−1^ region, corresponding to the stretching vibrations of the C–O–C glycosidic linkages [31], thereby confirming preservation of the cyclodextrin macrocyclic framework within the polymer structure. The most prominent features of the spectra, however, were bands associated with urethane linkages formed during the reaction of the hydroxyl groups of HPCD with the isocyanate groups of the crosslinkers. The band at approximately 1650 cm^−1^ was assigned to the stretching vibrations of the urethane carbonyl (C=O) group, whereas the bands in the 1550–1580 cm^−1^ range corresponded to N–H bending vibrations [32,33]. The intensity of these bands increased systematically with increasing molar excess of the crosslinking agent, indicating a controllable crosslinking process. In addition, the spectra of the polyurethane-based MIPs displayed absorption bands in the 1180–1280 cm^−1^ region, whose intensity correlated with the amount of crosslinker employed. These bands can be attributed to the stretching vibrations of C–O–C(O) groups within urethane fragments, with additional contributions arising from the C–O–C vibrations of the cyclodextrin glycosidic framework [34].

Comparative analysis of polymers prepared with different crosslinkers revealed distinct structural features. In the case of HPCD–TDI_MIP/NIP_ polymers, the band at approximately 1650 cm^−1^ was significantly influenced by stretching vibrations of the aromatic C=C bonds of the TDI moiety, resulting in a markedly higher absolute intensity compared to HPCD–HMD_MIP/NIP_ polymers. Conversely, the latter exhibited pronounced absorption bands at 2850 and 2950 cm^−1^, corresponding to the stretching vibrations of methylene groups within the saturated aliphatic segment of HMD. Overall, the FTIR data unequivocally confirm the successful formation of urethane crosslinks within the polymer matrix, preservation of the cyclodextrin monomer structure, and the structural individuality of the polymers determined by the nature of the diisocyanate crosslinker employed.

The structure of the hybrid Chit–HPCD–Genipin_MIP_ was monitored by FTIR spectroscopy at each stage of synthesis (Figure 5d–f). The spectrum of the Chit–HPCD conjugate exhibited characteristic HPCD absorption bands in the 1000–1100 cm^−1^ region, corresponding to the stretching vibrations of the C–O–C glycosidic linkages of the HPCD macrocycle. Direct confirmation of secondary amine formation resulting from covalent attachment of HPCD to chitosan was difficult due to overlap with the absorption bands of the primary amino groups of chitosan in the vicinity of 1550 cm^−1^ [35]. Nevertheless, because the synthesized conjugate was extensively purified by equilibrium dialysis to remove all non-bound HPCD molecules, the presence of cyclodextrin-specific bands in the FTIR spectrum provides clear evidence for the covalent incorporation of HPCD into the chitosan polymer backbone.

The spectra of both imprinted and non-imprinted polymers without template molecules crosslinked with genipin displayed an intense absorption band at approximately 1650 cm^−1^, corresponding to the stretching vibrations of the carbonyl groups of amide linkages formed during the reaction between genipin and the amino groups of chitosan. The appearance of this band is a characteristic indicator of successful crosslinking and confirms the formation of a three-dimensional polymer network in the synthesized hybrid materials. In general, the close similarity of the spectra obtained for both MIPs confirms the reproducibility of the polymer matrix composition regardless of the nature of the template molecule employed.

#### 2.2.2. IR Microscopy Mapping of Component Distribution in the Polymer Matrix

**Figure 6:**
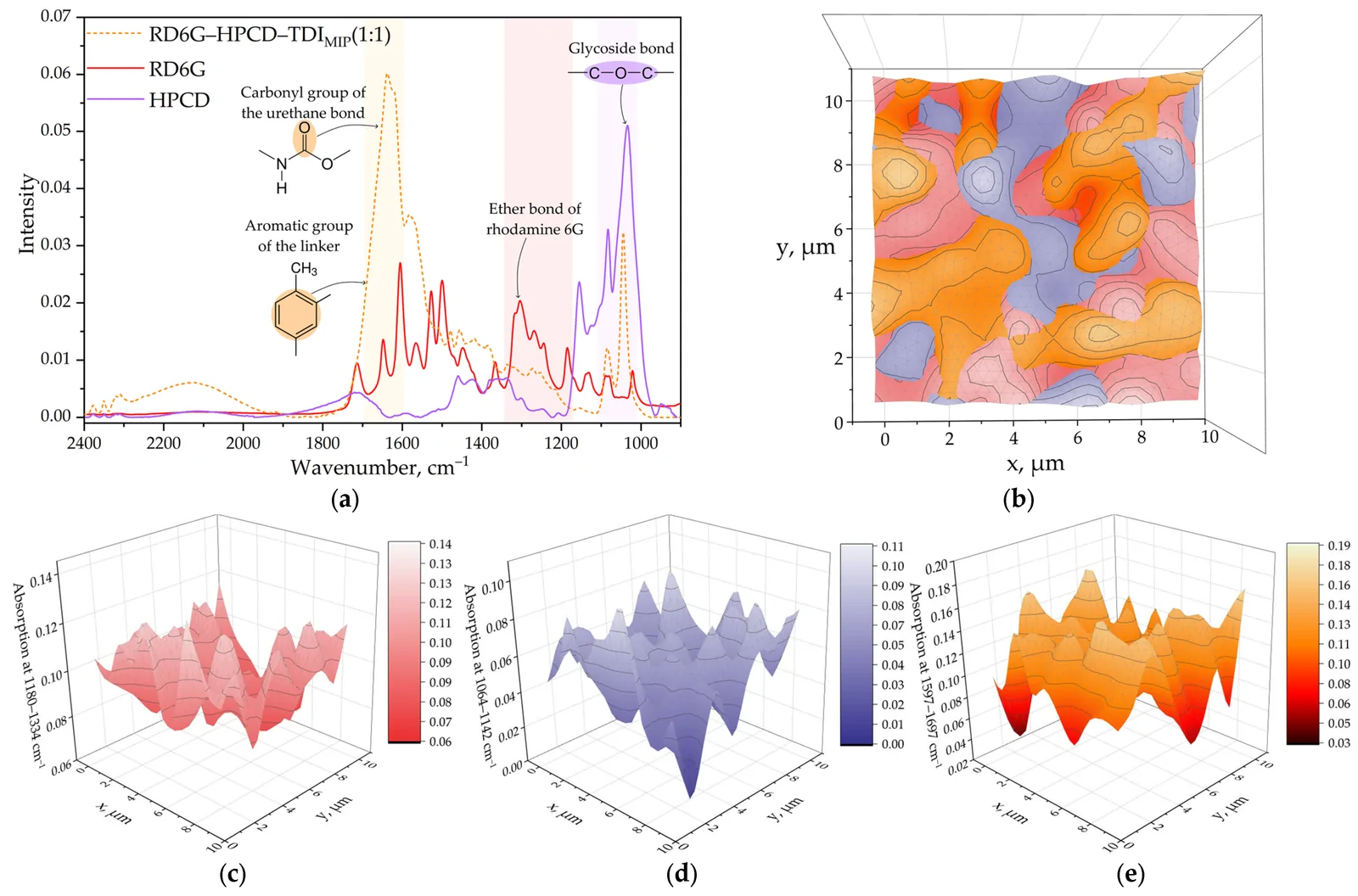
(**a**) FTIR spectra of HPCD, rhodamine 6G (RD6G) and RD6G’s MIP based on HPCD crosslinked with TDI in molar excesses of the linker 1:1 (RD6G–HPCD–TDI_MIP_ (1:1)); (**b**) superimposing absorption distribution maps of RD6G–HPCD–TDI_MIP_ (1:1) (above view); map of the absorption distribution of HPCD–TDI+ RD6G in the range of (**c**) 1180–1334 cm^−1^; (**d**) 1064–1142 cm^−1^; (**e**) 1597–1697 cm^−1^.

For a more detailed investigation of the spatial organization of the HPCD-based MIPs, absorption distribution maps of the characteristic FTIR bands were generated using FTIR microscopy, taking as an example the TDI-crosslinked MIP imprinted with rhodamine 6G (Figure 6). Rhodamine 6G was selected for this experiment because of its intense coloration, which allows its incorporation into the polymer particles to be visually monitored with the naked eye, thereby facilitating the assessment of distribution homogeneity (Figure 6b). The spectral regions of 1064–1142, 1180–1334, and 1597–1697 cm^−1^ were of particular analytical interest (Figure 6a), as these ranges correspond predominantly to the absorption of HPCD, rhodamine 6G, and urethane linkages, respectively, enabling visualization of the spatial localization of all components within the polymer matrix.

The generated absorption maps demonstrate a homogeneous distribution of the components throughout the polymer volume without the formation of pronounced domains. The regions attributed to rhodamine 6G (1180–1334 cm^−1^) partially overlap with areas enriched in HPCD (1064–1142 cm^−1^). At the same time, zones of local dye accumulation correlate with regions exhibiting enhanced absorption of urethane groups (1597–1697 cm^−1^), indicating the immobilization of template molecules within the polymer network formed by the crosslinker. These observations suggest that the imprinted cavities are distributed relatively uniformly throughout the polymer matrix and are formed in close proximity to both cyclodextrin moieties and urethane bridges, which provide their spatial stabilization [36].

### 2.3. Characterization of Synthesized Polymers

#### 2.3.1. Determination of the ζ-Potential of Synthesized Polymers

The ζ-potential values (obtained by the method dynamic light scattering, DLS) of the synthesized polymer materials were determined as an important colloidal characteristic, defined as the difference in electric potential between the slipping plane and the bulk solution, and serving as an indirect indicator of the colloidal stability of the particles [37]. The ζ-potential measurements revealed a pronounced dependence of the surface charge on both the nature of the template molecule and the polymer structure (Table 1). In general, NIPs exhibited relatively low absolute ζ-potential values, indicating the absence of strongly charged species associated with the polymer surface. In contrast, imprinting resulted in substantial changes in the electrokinetic properties of the materials, confirming the contribution of template molecules to the surface organization of the polymer matrix. The most pronounced effect was observed for fluorescein-imprinted MIPs. In both FL–HPCD–HMD_MIP_ (1:5) and FL–HPCD–TDI_MIP_ (1:1) systems, imprinting with fluorescein led to a significant shift in the ζ-potential toward negative values. The most negative ζ-potential among the cyclodextrin-based polyurethane MIPs was observed for FL–HPCD–TDI_MIP_ (1:1) (−22.0 mV). This observation is consistent with the proposed inclusion mechanism discussed above, in which the hydrophobic xanthene fragment is incorporated into the cyclodextrin cavity while the carboxyl group remains partially exposed to the aqueous environment, thereby contributing to the negative ζ-potential. The considerably more negative ζ-potential of the FL–HPCD–TDI_MIP_ (1:1) polymer compared with the corresponding FL–HPCD–HMD_MIP_ (1:5) material may additionally indicate stronger interactions between fluorescein and the aromatic TDI linker, resulting in more efficient retention of the template molecule near the polymer surface. In contrast, levofloxacin-imprinted polymers exhibited predominantly positive ζ-potentials. These findings agree with the proposed orientation of levofloxacin within the cyclodextrin cavity, where the protonated carboxyl-containing fragment is involved in host–guest interactions and contributes less to the surface charge. A similar trend was observed for chitosan-based materials: the non-imprinted Chit–HPCD–Genipin_NIP_ polymer displayed a positive ζ-potential (+12.1 mV), whereas imprinting with LV and especially FL resulted in a substantial decrease in surface charge. The stronger reduction observed for FL again supports its greater contribution to the electrostatic properties of the polymer surface. Overall, the ζ-potential data are in good agreement with the proposed modes of template incorporation and provide additional evidence for the formation of distinct recognition environments depending on the nature of the imprinted molecule.

#### 2.3.2. Determination of the Average Particle Size of Polymers

**Figure 7:**
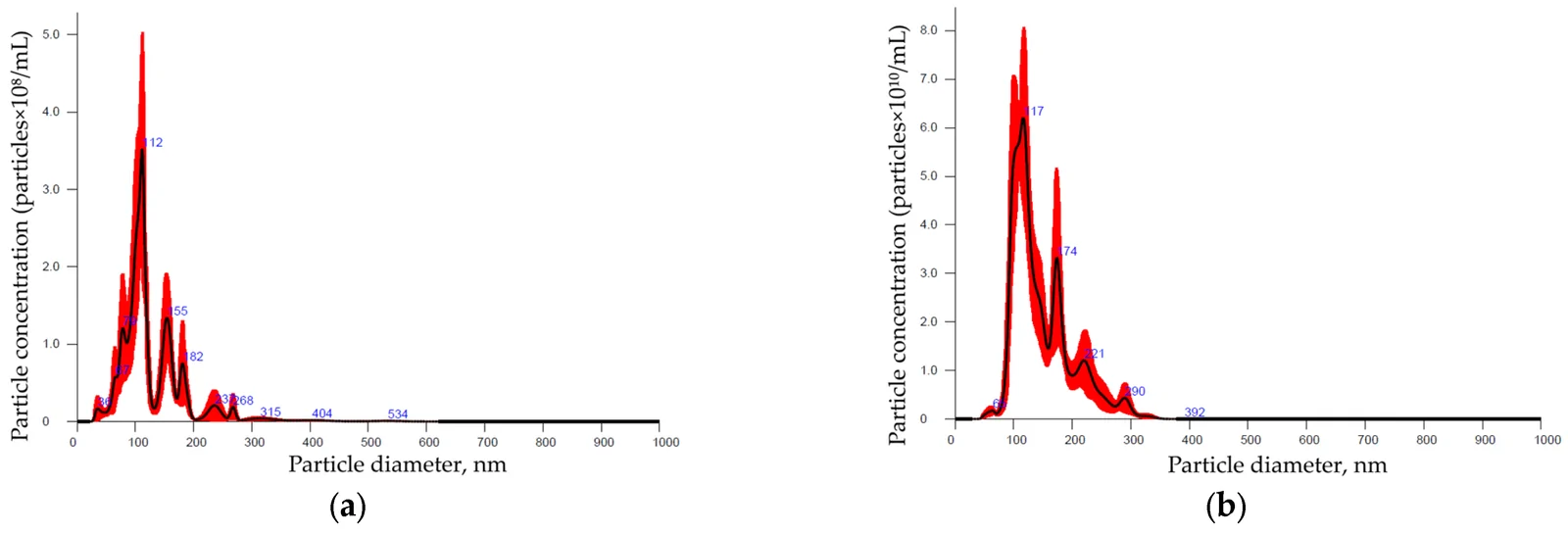
Particle size distribution diagram for (**a**) HPCD–HMD_NIP_ (1:5); (**b**) HPCD–TDI_NIP_ (1:1). The data were obtained using the NTA method. Additional NTA results are provided in the Supplementary Materials.

Nanoparticle tracking analysis (NTA) demonstrated that the synthesized polymers formed nanosized particles (Figure 7 and Figure S1). The HPCD-based polyurethane MIPs exhibited average hydrodynamic diameters ranging from 121 to 243 nm, whereas the chitosan-based polymers were somewhat larger, with average particle diameters of 301–352 nm. In general, molecular imprinting resulted in a moderate increase in particle size compared to the corresponding non-imprinted polymers, which may be attributed to the presence of template molecules during polymerization, leading to a less compact packing of the polymer network and, consequently, larger particles. The molecular weights of the HPCD-based polymers were 171–242 kDa, corresponding to degrees of polymerization of approximately 60–150, depending on the crosslinker type and its molar excess. These values are in good agreement with those reported for cyclodextrin-containing polyurethane materials [38]. In contrast, the Chit–HPCD–Genipin_MIP/NIP_ polymers exhibited substantially higher molecular weights (301–352 kDa), reflecting the larger molecular weight of the Chit–HPCD repeating unit and the formation of a more extended crosslinked polymer network.

For the HPCD-based polymers, the stoichiometry was estimated from the template losses during purification. Only 2–5% of the initially introduced template was removed as unbound material, indicating that approximately 95% remained associated with the polymer matrix. Since the initial HPCD–template complexes were prepared at a 1:1 molar ratio, the resulting effective stoichiometry was therefore approximated as 0.95:1:x (template:HPCD:crosslinker), where *x* represents the molar excess of the crosslinker. The stoichiometry of the chitosan-based systems is discussed separately below.

#### 2.3.3. Free Amino Group Titration with TNBS

**Figure 8:**
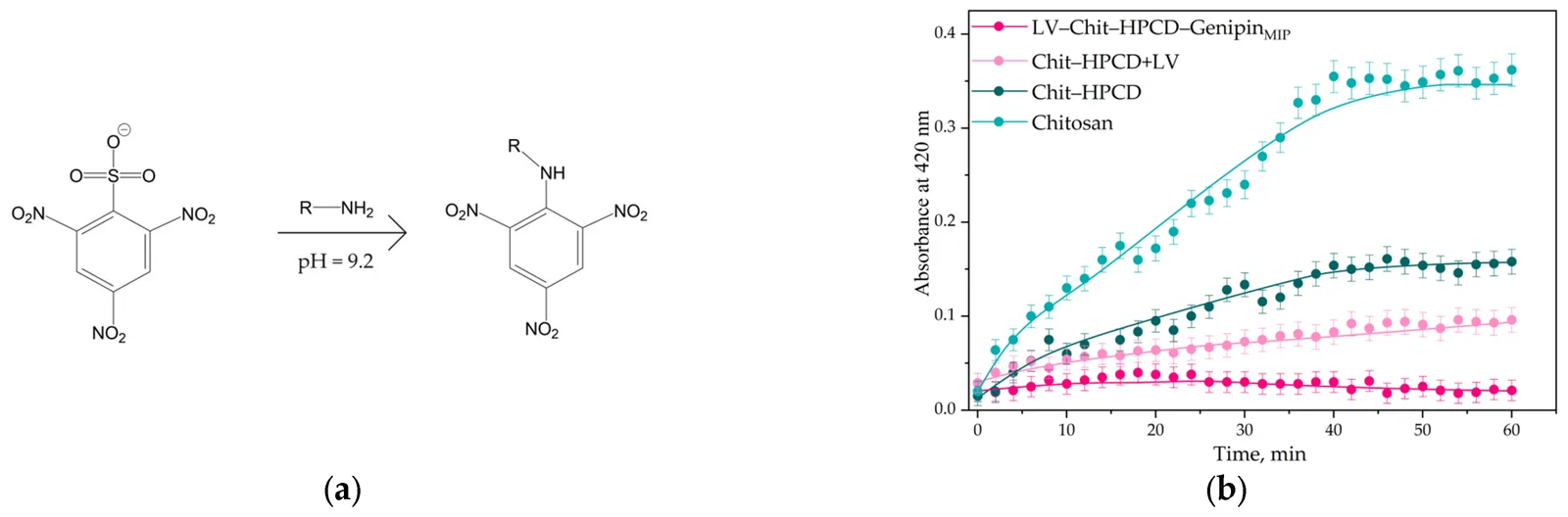
(**a**) Titration of free amino groups using 2,4,6-trinitrobenzenesulfonic acid (TNBS); (**b**) Kinetic curves at 420 nm for chitosan solution, chitosan with covalently linked HPCD (Chit–HPCD), non-covalent complex of LV with Chit–HPCD (Chit–HPCD + LV) and polymer LV–Chit–HPCD–Genipin_MIP_. Borate buffer solution. pH 9.2.

As an additional confirmation of the successful covalent grafting of HPCD onto chitosan following the tosylation step, the content of free amino groups was evaluated by TNBS titration (Figure 8). This analysis was performed to determine the fraction of amino groups that remained accessible after HPCD conjugation and subsequent reactions: a decrease in the TNBS-reactive amino groups relative to the initial chitosan was used to estimate the extent of HPCD grafting onto the chitosan backbone. Compared to native chitosan, the Chit–HPCD conjugate exhibited a pronounced decrease in the concentration of accessible primary amino groups, confirming that a substantial fraction of chitosan amino groups had participated in the reaction with tosylated HPCD, in agreement with our previous studies. Formation of the non-covalent Chit–HPCD + LV complex resulted in a further decrease in TNBS-reactive amino groups (The complex was formed in an acidic medium, then the titration was carried out at pH = 9.2). This behavior can be attributed to additional interactions between levofloxacin and the chitosan backbone. At pH = 3, the carboxyl group of LV (*pK_a_* = 5.5 [39]) is predominantly protonated, whereas chitosan amino groups (*pK_a_* = 6.0–7.3 depending on the molecular weight of the polymer and the degree of deacetylation [40,41]) are largely present in the NH_3_^+^ form. Under acidic conditions, protonation of chitosan amino groups may promote interactions with LV, while hydrogen bonding, hydrophobic interactions with the polymer backbone, and, potentially, ion-pair interactions arising from charge redistribution upon association with the polycationic matrix may further stabilize the polymer–LV complex [42]. Similar combinations of electrostatic and hydrogen-bonding interactions have been reported for chitosan-based adsorbents toward pharmaceutical compounds and organic pollutants [43]. Following genipin crosslinking to form LV–Chit–HPCD–Genipin_MIP_, the concentration of TNBS-accessible amino groups decreased to nearly zero, demonstrating that the remaining primary amino groups were efficiently consumed during network formation.

For the chitosan-based polymers, TNBS titration indicated approximately 16 HPCD residues per chitosan chain. Since 20–25% of the initially introduced template was removed during washing, approximately 75–80% remained associated with the polymer matrix. Accordingly, the effective stoichiometry was estimated as Chit:HPCD:template ≈ 1:16:12–13.

#### 2.3.4. AFM-Based Study

**Figure 9:**
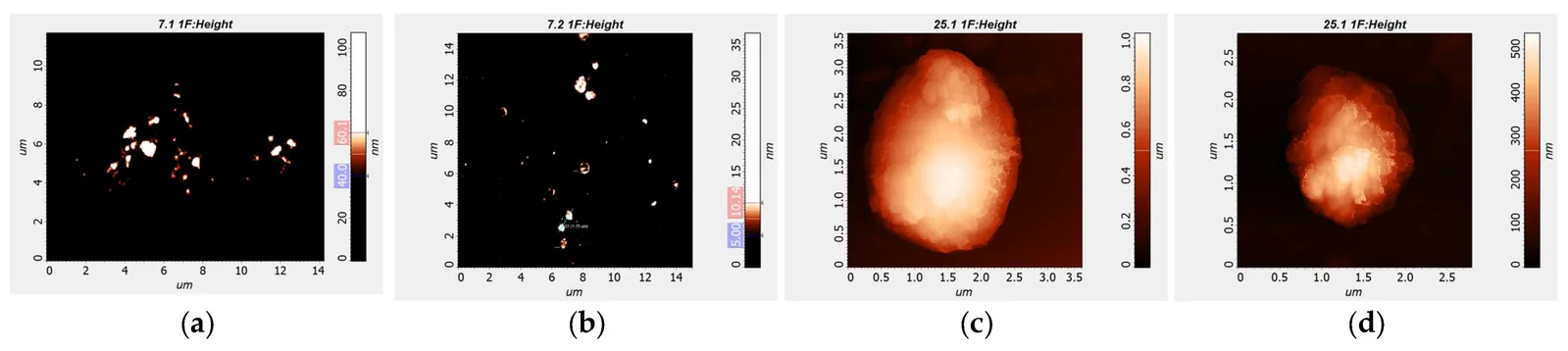
AFM photographs of (**a**) HPCD–TDI_NIP_ (1:1); (**b**) LV–HPCD–TDI_MIP_ (1:1); (**c**) HPCD–HMD_NIP_ (1:1); (**d**) LV–HPCD–HMD_MIP_ (1:5).

AFM was employed as a complementary technique to characterize the particle morphology, size distribution, and surface organization of the synthesized polymers. The AFM images (Figure 9) revealed relatively uniform individual particles with characteristic dimensions of approximately 200–350 nm, which are somewhat larger than the values obtained by NTA. This difference is attributable to the methodological features of AFM, particularly particle deposition and drying on the substrate, which can promote particle flattening, aggregation, and an apparent broadening of the measured lateral dimensions. A fraction of the particles was present in an aggregated state; representative images of such larger aggregates are shown in (Figure 9c,d), where the individual particle boundaries and surface organization can nevertheless be distinguished. Analysis of the corresponding surface profiles (Figure S2, Supplementary Materials) further indicated that the particles possess an approximately spherical morphology and revealed nanoscale surface depressions.

#### 2.3.5. SEM Analysis

SEM analysis revealed predominantly homogeneous polymer particles with relatively uniform morphology and dimensions, although some particles were present as aggregates. The observed morphology is consistent with the formation of crosslinked HPCD-based polymer particles and is consistent with the data obtained by the AFM method. SEM image of HPCD–HMD_NIP_ (1:5) are provided in the Supplementary Materials (Figure S3).

#### 2.3.6. BET Analysis

For both imprinted and non-imprinted chitosan-based polymers (LV/FL–Chit–HPCD–Genipin_MIP_ and Chit–HPCD–Genipin_NIP_), the pore size after removal of the template molecules was approximately 15–30 nm, corresponding to a specific surface area of approximately 3.5–5.0 m^2^/g. Upon interaction with LV or fluorescein, nitrogen adsorption was no longer detected, indicating that the pores became occupied by the analytes. Similarly, the pores of both imprinted and non-imprinted HPCD-based polymers were approximately 10–25 nm, while interaction with LV or fluorescein resulted in the disappearance of nitrogen adsorption, indicating pore occupation.

### 2.4. MIP’s Sorption Characteristics Studies

#### 2.4.1. Effect of pH and Crosslinker Content on the Sorption Properties of MIPs

Investigation of the effect of pH medium on the sorption properties of the synthesized MIPs is of particular importance, as this parameter governs the ionization state of both the functional groups of the polymer matrix and the analyte molecules, thereby directly affecting the efficiency of their interactions. In the context of analytical applications, pH optimization enables sample preparation under conditions that maximize sorption efficiency.

**Figure 10:**
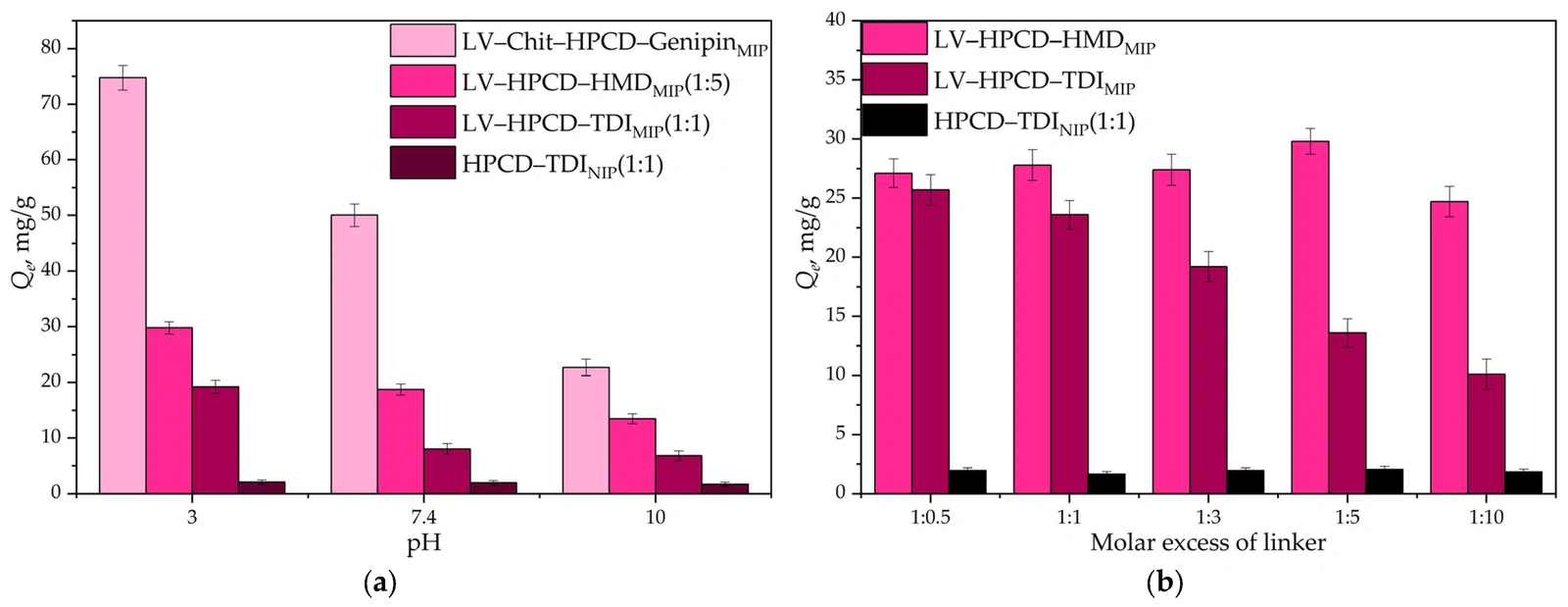
Diagrams illustrating (**a**) the dependence of the sorption capacity on pH for the levofloxacin’s MIPs and NIP as a control; (**b**) the dependence of the sorption capacity on the molar excess of the crosslinking agent in the polymer for the levofloxacin’s MIPs NIP as a control.

To evaluate the pH dependence of sorption, three model pH values were selected: 3.0, 7.4, and 10.0, corresponding to acidic, neutral, and alkaline media, respectively. NIPs of identical composition (without template-molecule) were used as controls, allowing simultaneous assessment of both the overall sorption capacity of the matrix and the contribution of molecular imprinting to analyte binding. As the parameter characterizing sorption performance, the equilibrium sorption capacity (*Q_e_*) was used, defined as the amount of analyte retained by the polymer matrix at equilibrium per unit mass of sorbent (mg/g). The experimental results demonstrated that the highest sorption capacity for all polymer types (LV–HPCD–HMD_MIP_, LV–HPCD–TDI_MIP_, and LV–Chit–HPCD–Genipin_MIP_) was achieved at pH 3.0 (Figure 10a). For LV–Chit–HPCD–Genipin_MIP_ the optimal sorption pH was likewise 3.0, but it is worth noting that this system exhibited a substantially higher sorption capacity than the HPCD-based polymers crosslinked with diisocyanates, reaching 74.5 mg/g at pH 3.0. This enhanced sorption may be associated with the additional contribution of the chitosan matrix and the hydrogen-bonding, hydrophobic, and other interactions discussed above. In contrast, the lowest sorption for all polymers was observed at pH 10, where deprotonation of the LV carboxyl group increases its negative charge, disfavouring penetration into the hydrophobic HPCD cavity and interactions with the hydrophobic regions of the crosslinker. The obtained sorption capacities are relatively high and comparable to those reported in the literature. For example, the authors of the study [44] reported cyclodextrin-based polymeric adsorbents exhibiting a sorption capacity of 23.3 mg/g toward pimavanserin, an atypical antipsychotic drug used for the treatment of Parkinson’s disease psychosis.

An important technological parameter governing the structure and sorption performance of MIPs is the molar excess of the crosslinking agent, which directly controls the crosslinking density of the polymer matrix and, consequently, the accessibility of the imprinted cavities to analyte molecules. For HPCD–HMD_MIP_ polymers, variation in the crosslinker-to-monomer ratio had little influence on sorption efficiency, with all investigated samples exhibiting sorption capacities of approximately 27 mg/g (Figure 10b). This behavior may be attributed to the flexibility of the aliphatic hexamethylene segment of HMD, which preserves sufficient chain mobility and accessibility of binding sites even at high crosslinking densities. In contrast, HPCD–TDI_MIP_ polymers exhibited a pronounced dependence of sorption capacity on the molar excess of the crosslinker. Increasing the TDI excess from 0.5 to 10 resulted in a more than twofold decrease in sorption capacity, from 25.7 to 10.1 mg/g. This trend can be attributed to the rigidity of the aromatic TDI fragment: increasing its content leads to the formation of an excessively dense and rigid polymer network, thereby hindering analyte binding. Based on the overall sorption performance, HPCD–HMD_MIP_ and HPCD–TDI_MIP_ synthesized at molar ratios of 1:5 and 1:1, respectively, were identified as the optimal formulations. Since these optimized formulations were employed in all subsequent experiments, the indication of the crosslinker molar ratio has been omitted from the polymer acronyms throughout the remainder of the manuscript for the sake of brevity.

Following the identification of optimal synthesis and sorption conditions, analogous studies were performed for MIPs imprinted with fluorescein (Table 2). For all investigated polymer systems, sorption capacities toward FL were substantially higher than those observed for LV. The most pronounced effect was observed for the HPCD–TDI_MIP_ polymer, whose sorption capacity reached 135.5 mg/g, whereas the corresponding levofloxacin-imprinted MIP exhibited a capacity of only 23.6 mg/g. The enhanced sorption efficiency toward fluorescein can be attributed to several factors. First, the rigid planar aromatic structure of fluorescein promotes additional π–π interactions with the aromatic moieties of TDI. Furthermore, fluorescein may participate in cooperative supramolecular interactions with cyclodextrins, potentially inducing a more ordered organization of cyclodextrin aggregates and facilitating the formation of geometrically well-defined recognition sites. In addition, the larger conjugated π-system and greater hydrophobicity of fluorescein contribute to stronger noncovalent interactions with the polymer matrix. For the chitosan-based particles, the lower *pKa* of the fluorescein carboxyl group (*pKa* = 4.31 [45]) compared to LV may make ion–dipole and, potentially, charge-assisted ionic interactions with protonated chitosan more favorable than for LV, particularly under acidic conditions. These results demonstrate the significant influence of template structure on the sorption properties of MIPs and highlight the potential of HPCD-based materials for the development of high-capacity sorbents.

#### 2.4.2. Selectivity Studies of the MIPs

To evaluate the selectivity of the synthesized MIPs, competitive sorption experiments were performed under model conditions. Levofloxacin sorption was studied in the presence of its structural aromatic analog—fluorescein and vice versa, enabling quantitative assessment of the ability of the polymer matrix to discriminate between structurally related molecules. It should be noted that the non-imprinted polymers without template molecules used as controls exhibited selectivity coefficients close to unity, indicating the absence of preferential analyte binding.

The calculated selectivity coefficients for levofloxacin-imprinted MIPs varied substantially depending on polymer composition. For HPCD–HMD_MIP_, selectivity coefficients ranged from 6 to 7 depending on the molar excess of the crosslinking agent. The hybrid LV–Chit–HPCD–Genipin_MIP_ polymer exhibited a selectivity coefficient of 14, whereas the highest selectivity was observed for the LV–HPCD–TDI_MIP_ (1:1) molar ratio, with a selectivity coefficient reaching 28. This high value may be attributed to the optimal crosslinking density achieved under these conditions, where the rigid aromatic linker most effectively preserves the geometry of imprinted cavities highly complementary to the template molecule while restricting access to structurally distinct analogs.

Similar experiments conducted with fluorescein-imprinted MIPs revealed even higher selectivity coefficients. In particular, the FL–HPCD–TDI_MIP_ (1:1) polymer exhibited a selectivity coefficient of 84.1, whereas values of 54.9 and 32.7 were obtained for FL–HPCD– HMD_MIP_ (1:5) and FL–Chit–HPCD–Genipin_MIP_ polymers, respectively. These results indicate the formation of highly specific recognition sites, likely resulting from a combination of factors, including the greater structural rigidity and extended aromatic π-system of fluorescein, which enhance hydrophobic and π–π interactions with aromatic TDI fragments, as well as improved geometric complementarity between the imprinted cavities and the template molecule. Moreover, the high conformational rigidity of fluorescein facilitates more accurate fixation of its spatial arrangement during polymerization, leading to the formation of binding sites with enhanced selectivity.

#### 2.4.3. Sorption Performance Under Repeated Loading Cycles

**Figure 11:**
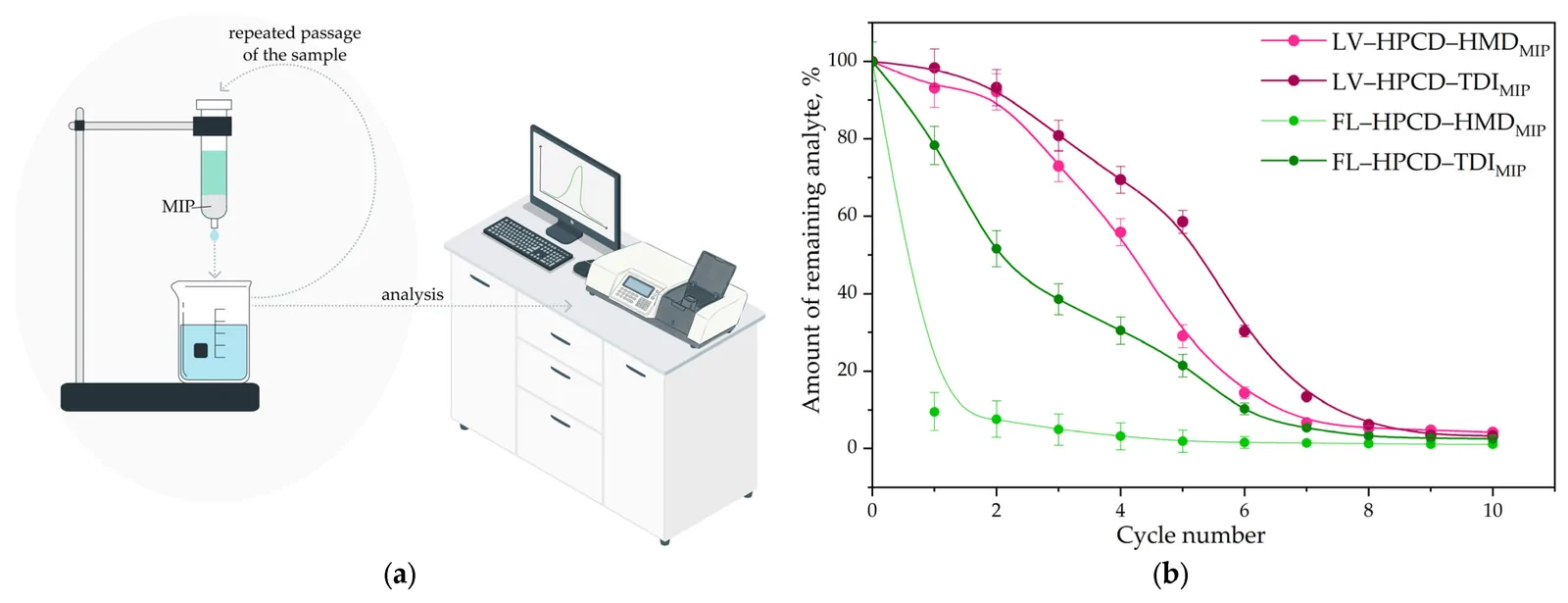
(**a**) Scheme of the sorption column with repeated passage of the analyte solution; (**b**) curves of changes in the amount of analyte in the analyzed solution depending on the number of cycles of passage through the MIPs.

To evaluate the dynamic binding characteristics, analyte solutions were repeatedly passed through columns packed with purified MIPs (Figure 11a). The solution was passed through the column under gravity at a natural flow rate of approximately 30 drops/min, without external pressure. The relatively low flow rate was intentionally maintained to increase the contact time between the analyte and the MIP recognition sites, as lower flow rates have been reported to improve the performance of MIP-based columns [46]. The results demonstrated that extraction efficiency strongly depended on both the nature of the template molecule and the structure of the polymer matrix (Figure 11b). For LV–HPCD–HMD_MIP_, a gradual decrease in analyte concentration was observed, with more than 70% of levofloxacin removed after six cycles and less than 5% of the initial amount remaining in solution after ten cycles. A similar trend was observed for LV–HPCD–TDI_MIP_, although the extraction rate was slightly lower.

In the case of fluorescein, extraction efficiency was significantly higher. After only two cycles through the FL–HPCD–HMD_MIP_ column, less than 10% of the analyte remained in solution, while nearly complete extraction was achieved after eight cycles for FL‒HPCD–TDI_MIP_. These findings are consistent with the high sorption capacities and selectivity coefficients observed for fluorescein-imprinted MIPs and indicate stronger and more numerous interactions between the xanthene dye molecules and the recognition sites of the polymer matrix. The slower extraction of levofloxacin is likely associated with its lower molecular rigidity and, consequently, the reduced geometric complementarity of the corresponding binding sites.

In addition, the reusability of all synthesized MIPs was evaluated over at least ten consecutive sorption–desorption cycles, demonstrating a decrease in sorption efficiency of no more than 3%, which confirms their excellent regeneration capability and highlights their considerable practical potential for repeated analytical and biomedical applications.

#### 2.4.4. Desorption Kinetics of Template Molecules

**Figure 12:**
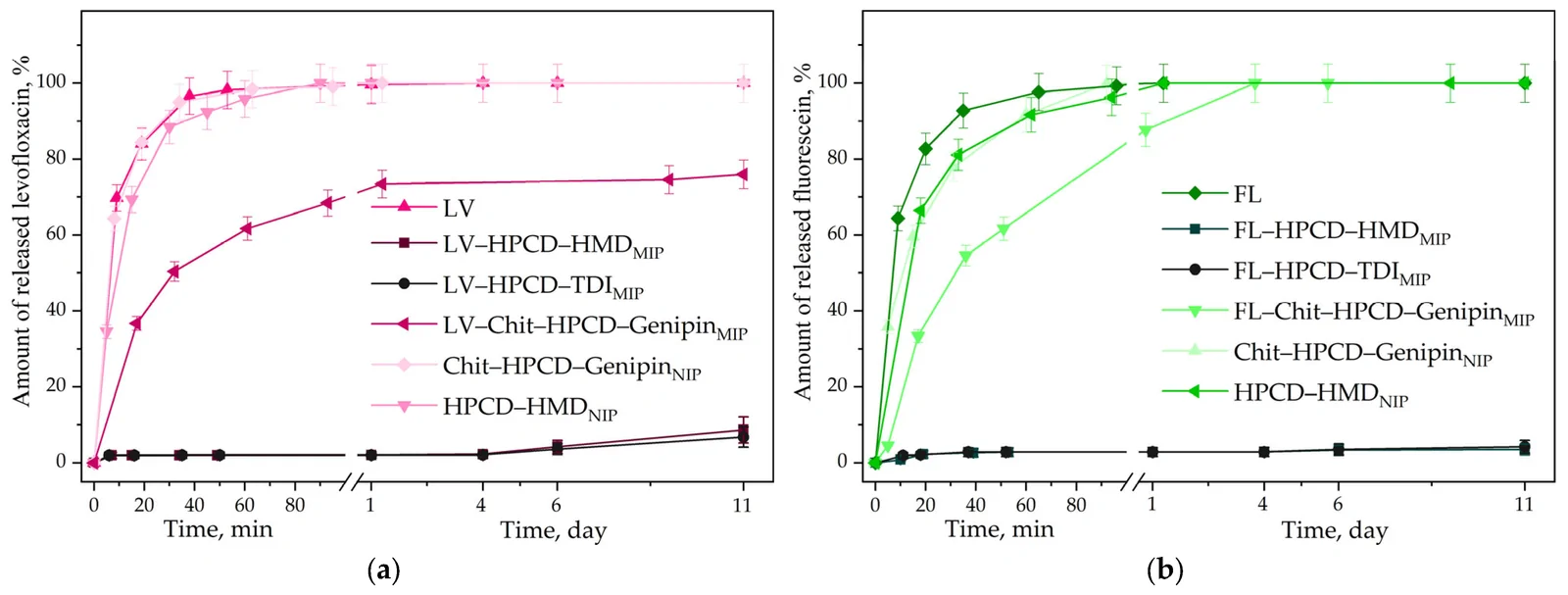
Curves of changes in the amount of (**a**) levofloxacin and (**b**) fluorescein in the external solution depending on the time of release without a carrier, from MIPs or non-imprinted polymers, pH = 10.

The release kinetics were evaluated to determine the strength of analyte retention within the polymer matrix, estimate the fractions of strongly retained and weakly associated templat. Investigation of the release kinetics of template molecules from the MIPs revealed substantial differences between polyurethane-based HPCD polymers and genipin-crosslinked chitosan–HPCD hybrid materials (Figure 12). Chit–HPCD–Genipin_MIP_ samples exhibited rapid analyte release, with more than 60% of both levofloxacin and fluorescein released within the first 30–60 min, while complete fluorescein release was achieved within 24 h. In contrast, polyurethane MIPs displayed markedly slower release kinetics. For the HPCD–HMD_MIP_, no more than 10% of the template molecule was released throughout the entire observation period. The virtually complete absence of release from HPCD–TDI_MIP_ indicate exceptionally strong interactions between the analyte and the recognition sites under these conditions. Notably, the fraction released during the initial ~10 min is considered the weakly associated or unbound template, whereas the remaining fraction is more strongly retained within the polymer matrix. Thus, approximately 95–98% of the template for HPCD-based MIPs and ~80% for chitosan-based MIPs were considered polymer-associated and used for estimating the effective template stoichiometry.

The release kinetics demonstrated that the imprinted polymers retained the analytes strongly within their matrices. Notably, even under pH conditions corresponding to minimal sorption and, therefore, favorable conditions for desorption, the release proceeded slowly. This indicates strong analyte–matrix interactions and suggests that simple pH adjustment may be insufficient for efficient sorbent regeneration. More effective regeneration may require the use of appropriate organic solvents, such as alcohol-containing systems, as well as controlled temperature treatment to facilitate analyte desorption from the polymer matrix.

### 2.5. Investigation of MIPs Binding Constants with Template Molecules

**Figure 13:**
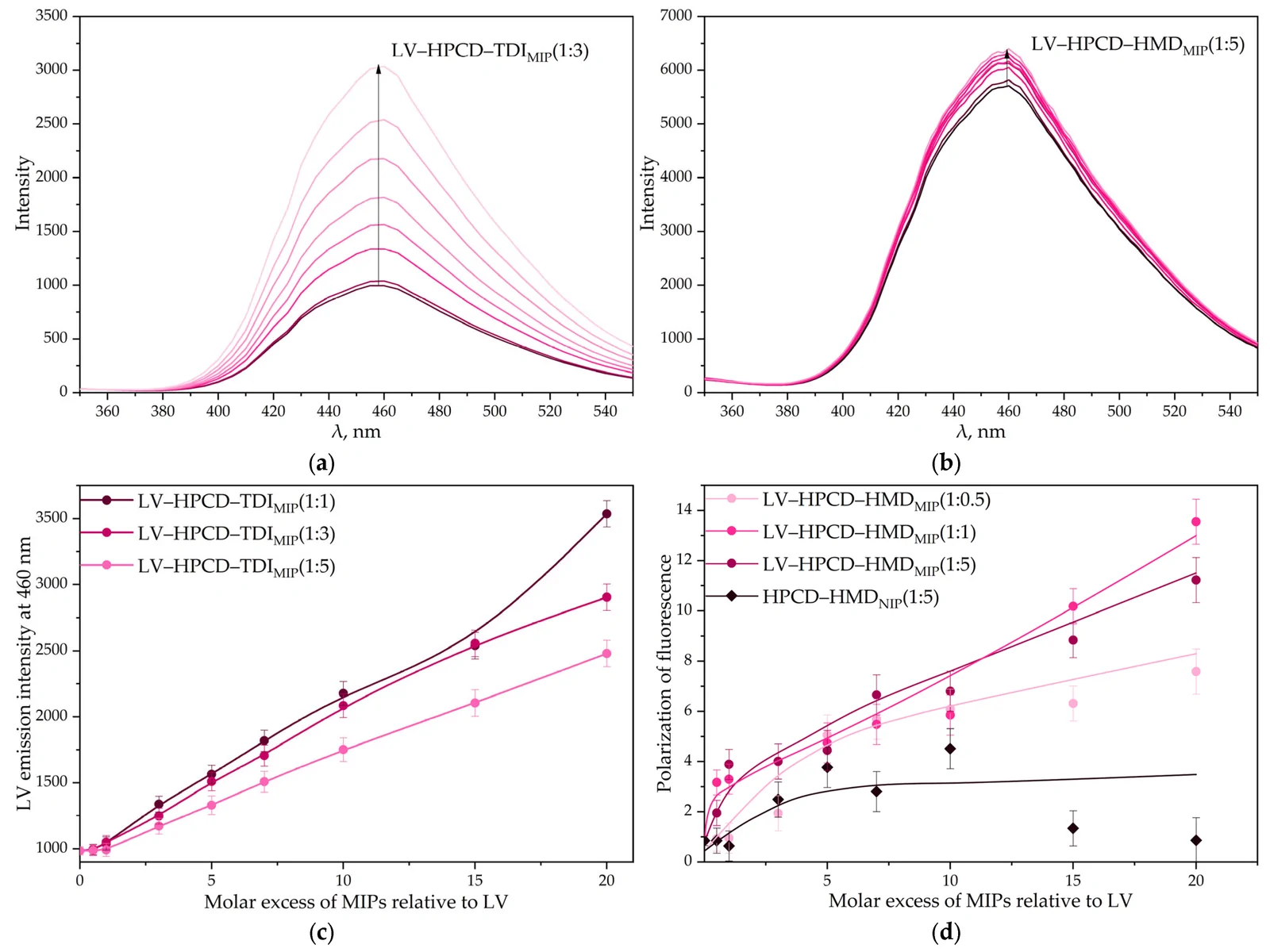
Emission spectra of levofloxacin fluorescence with an increase in the molar excess of the MIPs in a complex with (**a**) LV–HPCD–TDI_MIP_ (1:3); (**b**) LV–HPCD–HMD_MIP_ (1:5); (**c**) the curve of the change in the maximum emission of levofloxacin at 460 nm, depending on the molar excess of MIPs; (**d**) the polarization of levofloxacin fluorescence in the complex with MIPs or non-imprinted polymers (HPCD–HMD_NIP_ (1:5)), depending on the increase in the molar excess of the polymer carrier.

To quantitatively characterize the affinity of the synthesized MIPs (after purification from the template molecule) toward the target analytes, dissociation constants of the polymer–analyte complexes were determined by fluorescence spectroscopy. It was established that the interaction of analytes with the polymer matrices is accompanied by a change in fluorescence emission intensity (Figure 13a,b), which enabled quantitative description of the binding process using the Hill’s equation and calculation of the corresponding dissociation constants (Table 3). The *K_dis_* values obtained for all polymer systems were 1–2 orders of magnitude lower than that of monomeric HPCD indicating a substantial enhancement of binding affinity as a result of polymerisation and the formation of imprinted cavities. Interestingly, the monomeric HPCD exhibited comparable affinities toward both levofloxacin and fluorescein, with dissociation constants of 9.6 × 10^−4^ and 7.5 × 10^−4^ M, respectively, indicating that inclusion complex formation alone does not provide pronounced discrimination between these analytes. In contrast, after polymerization and imprinting, a substantial difference in binding affinity emerged. This effect was particularly evident for the HPCD–HMD_NIP_ (1:5) polymer, for which the *K_dis_* value toward FL was nearly one order of magnitude lower than that observed for LV. Such behavior may be attributed to additional electrostatic interactions involving the fluorescein carboxyl group, which is expected to remain partially exposed outside the cyclodextrin cavity. Residual amino groups formed through hydrolysis of unreacted isocyanate functionalities of the linker [47], present in excess during polymer synthesis, may provide additional binding sites for fluorescein. The combination of inclusion complexation and these secondary ionic interactions likely contribute to the enhanced affinity of the polymer matrix toward FL compared to LV.

For HPCD–HMD_MIP_ the lowest *K_dis_* value was observed at a crosslinker molar ratio of 1:5, reflecting the strongest analyte binding and consistent with the results of the sorption experiments. For HPCD–TDI_MIP_ the sample prepared at a molar ratio of 1:1 exhibited the highest affinity, with a *K_dis_* of 1.2 × 10^−5^ M. The low dissociation constant observed for this material may additionally reflect the contribution of aromatic interactions between the TDI-derived linker fragments and the aromatic regions of the template molecules. Such interactions are expected to be particularly important for those recognition sites formed from HPCD–template complexes involving external association mechanisms, where the template molecule remains accessible outside the cyclodextrin cavity during polymerization. Consequently, external association complexes may play a crucial role in molecular imprinting by providing additional fixation points that enhance both the affinity and structural fidelity of the resulting recognition sites. The dissociation constants obtained are of practical significance as a selection criterion for identifying the most promising candidates for further application, and may additionally serve as a basis for predicting sorption performance in real analytical and biomedical systems.

To directly assess the molecular selectivity of the imprinted recognition sites, the affinity of the LV-imprinted MIPs toward FL (and vice versa) was evaluated as a structurally distinct analog. The resulting complexes were characterized by substantially higher *K_dis_* values, reaching 10^−2^–10^−1^ M, indicating markedly weaker binding and reflecting the low affinity of the imprinted cavities toward a structurally dissimilar analog. An analogous pattern was observed for fluorescein-imprinted MIPs: these materials exhibited low *K_dis_* values with respect to their own template (1.1 × 10^−4^–1.2 × 10^−5^ M) and considerably higher values upon complexation with levofloxacin. Particularly pronounced selectivity was observed for the FL–HPCD–TDI_MIP_ (1:1) polymer, for which the *K_dis_* of the complex with FL was 1.2 × 10^−5^ M, whereas the corresponding value for LV exceeded this by more than four orders of magnitude, reaching 2.6 × 10^−1^ M. These findings are consistent with the results of the sorption experiments, which demonstrated high selectivity coefficients and substantially greater sorption capacity toward the respective template molecules. Taken together, the dissociation constants confirm that the formation of imprinted cavities not only enhances the overall binding affinity of the polymer matrix, but also gives rise to pronounced molecular selectivity, arising from the geometric and functional complementarity of the recognition sites to the corresponding analytes.

Further evidence for the efficiency of analyte binding to the polymer matrices was provided by fluorescence polarization measurements of LV, which allow assessment of changes in molecular mobility upon complexation. It is well established that an increase in fluorescence polarization reflects the restriction of rotational and translational mobility of a fluorophore upon its incorporation into a larger complex [48]. Accordingly, an increase in polarization is indicative of analyte inclusion within the imprinted cavities of the MIP and the formation of a stable polymer–analyte complex. It was demonstrated that upon interaction of LV with the MIP, a progressive increase in fluorescence polarization was observed with increasing molar excess of the polymer carrier, consistent with the restriction of analyte mobility upon inclusion within the imprinted cavities of the matrix (Figure 10b). In addition to providing qualitative evidence of complex formation, fluorescence polarization data can be used for quantitative evaluation of binding parameters. Accordingly, dissociation constants were estimated from the polarization dependence on polymer concentration for levofloxacin-containing systems. The obtained values were of the same order of magnitude as those determined independently earlier (Table 3). Such agreement provides additional confirmation of the reliability of the calculated binding constants. In the case of the non-imprinted control polymer, no analogous trend was observed, indicating the absence of specific binding and confirming the indispensable role of the imprinted recognition sites in template retention. Accordingly, LV–HPCD–TDI_MIP_ (1:1) can be considered the most selective material for levofloxacin, whereas FL–HPCD–TDI_MIP_ (1:1) is the most selective for fluorescein. Collectively, the fluorescence polarization data, together with the dissociation constants and the results of the sorption analysis, unambiguously confirm the specific character of the interactions between the synthesized MIPs and the target analytes, and validate the effectiveness of the molecular imprinting approach employed.

### 2.6. The Use of MIP to Extract Analytes from Real Objects

To evaluate the applicability of the synthesized MIPs for concentrating real analytical samples, fluorescein was selected as a model compound owing to its intense coloration and strong UV absorption, which enable straightforward quantitative determination of the residual analyte concentration by UV–Vis and fluorescence spectroscopy. Analysis of biological and food matrices generally requires extensive sample preparation to remove proteins, lipids, and other interfering components prior to analyte determination. This requirement represents one of the major limitations of conventional analytical approaches. Milk contains high concentrations of proteins, lipids, and colloidal particles capable of adsorbing analytes or interfering with instrumental detection, whereas blood plasma is an even more complex matrix comprising albumin, globulins, phospholipids, metabolites, amino acids, and numerous endogenous low-molecular-weight compounds that may compete with target analytes during extraction and analysis. As a consequence, conventional methods frequently require multistep sample preparation procedures involving protein precipitation, liquid–liquid extraction, solid-phase extraction, and matrix-cleanup stages prior to chromatographic determination. Such matrix effects are well documented and remain a significant challenge in bioanalytical and food analysis applications [49,50]. In the present study, however, the sample preparation protocol was intentionally minimized and consisted primarily of protein precipitation with ammonium sulfate followed by centrifugation to remove the resulting precipitate. Such a simplified procedure preserves substantially reducing the time and labor required for analysis.

Model samples of milk and blood plasma were prepared by spiking each matrix with fluorescein. It should be noted that a fraction of fluorescein was inevitably lost during the protein precipitation step. Therefore, immediately after sample preparation, the actual fluorescein concentration was determined using a calibration curve, and this experimentally determined concentration was taken as the initial value for all subsequent sorption experiments. The prepared samples were then repeatedly passed through columns packed with the synthesized MIPs, while the residual fluorescein concentration in the eluate was monitored after each cycle by UV–Vis or fluorescence spectroscopy.

**Figure 14:**
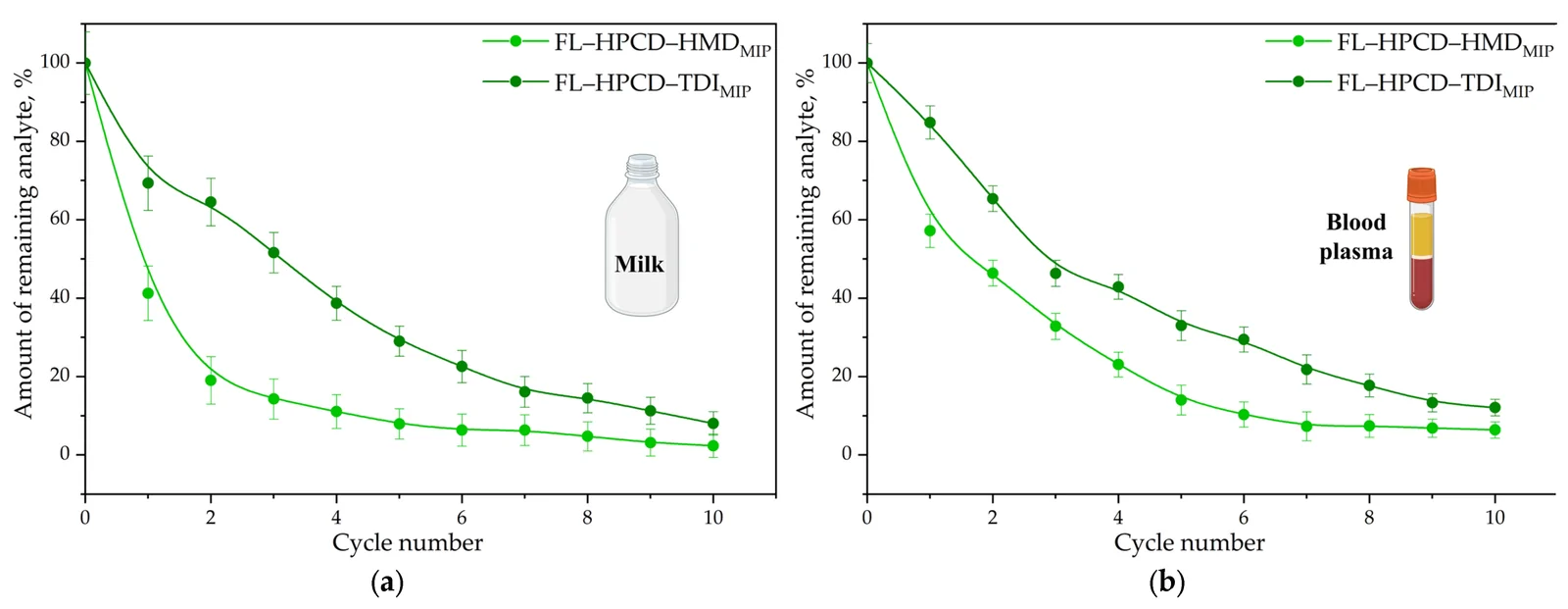
(**a**) Curves of changes in the amount of fluorescein in the analyzed solution of milk depending on the number of cycles of passage through the MIPs; (**b**) curves of changes in the amount of fluorescein in the analyzed solution of blood plasma depending on the number of cycles of passage through the MIPs.

Despite the presence of numerous competing compounds remaining in the pretreated matrices, both investigated MIPs demonstrated highly efficient extraction of fluorescein (Figure 14, Table 4 and Table 5). After ten consecutive sorption cycles, the residual analyte concentration in both milk and blood plasma decreased to below 10% of its initial value, corresponding to an extraction efficiency exceeding 90%. Such a high recovery achieved using only ten loading cycles clearly demonstrates that the synthesized MIPs retain their molecular recognition capability even in complex biological and food matrices and confirms their considerable potential for practical analytical applications as tools for concentration.

The extraction kinetics differed between the two polymer systems. The FL–HPCD–HMD_MIP_ sorbent exhibited faster fluorescein uptake during the initial sorption cycles, reaching recovery values of 92.1% and 86.0% from milk and blood plasma, respectively, after five cycles. In contrast, extraction by FL–HPCD–TDI proceeded more gradually, yielding recoveries of 71.0% and 66.9% after five cycles. However, after ten cycles both sorbents demonstrated high overall extraction efficiencies, with fluorescein recoveries reaching 97.6% and 91.9% for FL–HPCD–HMD_MIP_ and FL–HPCD–TDI_MIP_ from milk and 93.6% and 87.9% from blood plasma, respectively. These results confirm that the molecular recognition properties of the synthesized MIPs are largely preserved in complex biological matrices despite the presence of competing compounds. From a practical standpoint, efficient analyte extraction requires that the amount of analyte introduced into the sorption system remains below the maximum binding capacity of the polymer. In real samples, numerous competing species may partially occupy recognition sites or hinder mass transfer, thereby reducing the effective sorption capacity. Therefore, it is advisable to employ a sufficient excess of sorbent and to operate at analyte loads not exceeding approximately 70–90% of the experimentally determined sorption capacity. From an analytical perspective, HPCD–TDI_MIP_ remains particularly attractive because its high extraction efficiency is complemented by an exceptionally high selectivity coefficient (84.1), ensuring superior discrimination of the target analyte from structurally related interferents.

Quantitative analysis of the spiked samples (5 × 10^−5^ M fluorescein) demonstrated high analyte recoveries after correction for losses during protein precipitation. Comparable extraction efficiencies were obtained from milk and blood plasma, indicating that the residual matrix components had only a limited effect on molecular recognition. Because these matrices differ substantially in composition and generate considerable background signals from proteins, lipids, phospholipids, amino acids, and other endogenous compounds, analyte-free matrix controls were analyzed in parallel for baseline correction. Despite the simplified sample preparation, high extraction efficiencies were retained, demonstrating the robustness of the MIPs in complex matrices.

At lower fluorescein concentrations (10^−6^–10^−7^ M), fluorescence spectroscopy was used for quantitative detection, and the extraction efficiency remained above 80%. These results demonstrate that the developed MIPs can efficiently preconcentrate and enable subsequent determination of analytes over a broad concentration range, from at least 10^−4^ to 10^−7^ M using fluorescence/UV–Vis detection. Importantly, the analytical detection limit is determined not only by the sorbent but also by the sensitivity of the detection technique. Thus, further improvement in detection limits can be achieved by coupling the developed MIPs with more sensitive analytical platforms, such as chromatographic methods with mass-spectrometric detection. In the present study, UV–Vis and fluorescence spectroscopy were selected to carry out a wide screening of sorbens to select the most optimal and also because of their simplicity, accessibility, and rapid signal response. However, to understand the maximum sensitivity parameters for determining drugs or dyes in samples an additional experiment using HPLC–MS detection of levofloxacin in blood after MIP-based preconcentration was performed (calibration data are provided in the Supplementary Materials, Figure S4), yielding an extraction efficiency above 80% (at a concentration ~10^−6^ М).

## 3. Discussion

### Validation of Results with Literature Data

A comparison with previously reported MIPs (Table 6) demonstrates that the HPCD-based materials developed in this work are highly competitive and, in terms of selectivity, exceed most of the selected literature examples. For FL–HPCD–TDI_MIP_ (1:1), the sorption capacity reached 135.5 mg/g and the selectivity coefficient was 84.1, which is substantially higher than the values reported for sulpiride-, ofloxacin-, nalidixic acid-, and spiramycin-imprinted polymers, where selectivity typically ranged from approximately 2.6 to 5.36. Even the LV–HPCD–TDI_MIP_ (1:1) polymer demonstrated a selectivity coefficient of 27.5, exceeding most pharmaceutical MIPs included in the comparison. Although the Basic Blue 41 MIP showed a slightly higher sorption capacity of 144.8 mg/g, its recognition coefficient was maximum of 17.6, indicating that high capacity alone does not necessarily ensure molecular recognition. Thus, the main advantage of the present approach is the combination of high sorption capacity with pronounced molecular selectivity. The Chit–HPCD–Genipin_MIP_ systems showed lower selectivity than polyurethane HPCD–TDI_MIP_ materials, but their biocompatibility, biodegradability, and controlled-release behavior make them attractive for biomedical applications. Further work should focus on broadening the analyte scope, testing long-term regeneration, and adapting the materials for trace-level analysis.

**Table 6:** Comparison of the sorption characteristics of the obtained MIPs with the published data.

Polymer	Template Molecule	*Q_e_,* mg/g	Selectivity	Real Sample Application	Reference
LV–HPCD–HMD_MIP_ (1:5)	Levofloxacin	29.8	6.5	milk, blood plasma	This work
LV–HPCD–TDI_MIP_ (1:1)		23.6	27.5		
LV–Chit–HPCD–Genipin_MIP_		74.8	13.8		
FL–HPCD–HMD_MIP_ (1:5)		47.6	54.9	milk, blood plasma	This work
FL–HPCD–TDI_MIP_ (1:1)	Fluorescein	135.5	84.1		
FL–Chit–HPCD–Genipin_MIP_		84.7	32.7		
Methacrylic acid-based MIP	Albendazole	34.9	high (qualitative)	plasma and urine	[12]
Chitosan-based MIP	Remazol Red 3BS	18.5	21.02	not reported	[51]
Magnetic MIP based on cyclodextrin/Fe_3_O_4_	Tetracycline antibiotics	14.5	36.9	environmental water	[14]
*N*,*N*-dimethylacrylamide-based MIP	Nalidixic acid	13.8	not reported	urine	[52]
Methacrylic acid-based MIP	Spiramycin I	86.5	3.92	Purification of spiramycin I from fermentation broth by MIP chromatography	[53]
Itaconic acid-based MIP	Sulpiride	20.9 *	5.36 (against non-imprinted polymer)	rat serum	[54]
Carbon nanoparticles-based MIP	Ofloxacin	40.98	2.6 (against non-imprinted polymer)	Spiked seawater with fluoroquinolone antibiotics	[55]
Poly(ethylene terephatalate)-based MIP	Basic Blue 41	144.8	17.46 (vs. Acid Black 24) and 5.18 (vs. Acid Blue 129)	not reported	[56]
MAA/EGDMA-based MIP microspheres	Clenbuterol	not directly reported	>1 (against non-imprinted polymer)	not reported	[57]
MAA/EGDMA-based MIP	Malachite green	28.36	4.88–14.67	food-grade fish samples	[58]
MAA–EGDMA-based MIP	Levofloxacin	not reported	3.081 (against non-imprinted polymer)	Distilled, tap and river water	[59]
Hydrophilic ionic-liquid MIP	Levofloxacin	16.45	2.41 (against non-imprinted polymer)	Water, sediment	[60]

* Original value: 61.13 μmol/g; recalculated to mg/g using the molecular weight of sulpiride.

## 4. Materials and Methods

### 4.1. Materials

2-hydroxypropyl-β-cyclodextrin, chitosan oligosaccharide (*M_w_* = 5000, >90% deacetylated), genipin, 1,6-hexamethylenediisocyanate, levofloxacin, fluorescein, dimethyl sulfoxide, 2,4,6-trinitrobenzenesulfonic acid (TNBS), NaOH were all from Sigma-Aldrich (St. Louis, MO, USA). HCl and toluene diisocyanate were purchased from Reakhim (Moscow, Russia). Tablets for sodium-phosphate-buffered solution preparation (pH 7.4) were obtained from Pan-Eco (Moscow, Russia), Cat. №: Р071. Commercial whole milk (3.2% fat, “Domik v Derevne”, Moscow, Russia) and human plasma (Molecular Depot, San Diego, CA, USA).

### 4.2. Synthesis of HPCD-Based Polyurethane MIPs

Polyurethane molecularly imprinted polymers based on HPCD were synthesized according to a procedure developed in our previous studies [61,62] with substantial modifications to the complexation, polymerization, and purification steps. Briefly, the template molecule (levofloxacin, fluorescein, rhodamine 6G, or rifampicin) was mixed in water:DMSO solution (water:DMSO volume ratio of 1:1 in final solution) with HPCD at a molar ratio of 1:1 and incubated for 1 h at 37 °C under continuous stirring to allow the formation of a non-covalent HPCD–template complex. Separately, the required amount of HMD or TDI was dissolved in DMSO and the resulting linker solution was added dropwise to the reaction mixture under vigorous stirring. Polymerization was carried out for 2 h at 40 °C under continuous stirring. The molar excess of the crosslinker relative to HPCD was varied according to the experimental design. After completion of the reaction, the obtained polymers were purified by dialysis (cut-off 12 kDa molecular-weight membrane, Serva, Heidelberg, Germany) at room temperature by repeated washing with ethanol followed by alkaline aqueous solution (pH 10) to remove unreacted reagents and template molecules retained within the polymer matrix. The purification procedure was continued until no absorption bands attributable to the template molecules were detected in the UV–Vis spectra of the washing solutions. The polymers were then extensively washed with distilled water. Non-imprinted polymers were prepared using an identical protocol in the absence of template molecules.

### 4.3. Synthesis of Hybrid MIPs Based on Chitosan and HPCD

Hybrid chitosan–HPCD MIPs were synthesized through covalent attachment of HPCD to chitosan followed by template-assisted crosslinking with genipin. Initially, tosylated HPCD was prepared by dissolving HPCD (4 mmol) in 20 mL of distilled water. A solution of NaOH (3 mmol in 2 mL H_2_O) was added dropwise under continuous stirring, followed by the dropwise addition of a solution of p-toluenesulfonyl chloride (4 mmol in 3 mL DMSO). The reaction mixture was stirred continuously at room temperature for 3 h.

Subsequently, the pH of the reaction mixture was adjusted to 4.0 using hydrochloric acid, and the solution was heated to 90 °C. Chitosan (500 mg) was then added, and the reaction was carried out for 2 h at 90 °C under continuous stirring. The reaction mixture was subsequently incubated for an additional 10 h at 37 °C with continuous stirring. As a result, a yellowish gel-like product was formed. The obtained chitosan–HPCD conjugate was purified by equilibrium dialysis for several hours to remove low-molecular-weight impurities and unreacted reagents. After dialysis, the polymer was additionally washed with hot alcohol (~50 °C) and subsequently with distilled water to remove strongly retained template molecules from the polymer matrix. The purified material was concentrated by air drying.

The content of cyclodextrin moieties in the chitosan–HPCD conjugate was determined by FTIR spectroscopy. Based on the calculated concentration of cyclodextrin units, an equimolar amount of the template molecule was added to the conjugate solution and incubated for 1 h at 37 °C under continuous stirring to form a non-covalent polymer–template complex. Subsequently, a genipin solution prepared in a 1:1 (*v*/*v*) mixture of sodium phosphate buffer and ethanol was added to the reaction mixture. The system was thoroughly mixed and incubated overnight at 37 °C under continuous stirring to allow the formation of a crosslinked three-dimensional polymer network. The resulting molecularly imprinted polymers were purified by equilibrium dialysis until complete removal of unreacted reagents and non-bound template molecules.

Non-imprinted control polymers (NIP) were synthesized according to the same procedure in the absence of template molecules.

### 4.4. FTIR-Spectroscopy

Fourier-transform infrared (FTIR) spectroscopy was performed according to a procedure based on our previous studies [63,64] with modifications to sample preparation and measurement conditions. FTIR spectra were recorded using a Tensor 27 spectrometer (Bruker, Ettlingen, Germany) equipped with a liquid nitrogen-cooled MCT detector and a single-reflection ZnSe attenuated total reflectance (ATR) accessory (Bruker, Ettlingen, Germany). Prior to analysis, 5 μL of a concentrated aqueous solution of the samples was deposited onto the ATR crystal and dried at 37 °C to constant weight. Spectra were collected in triplicate in the range of 3000–900 cm^−1^ with a spectral resolution of 1 cm^−1^ and 70 accumulated scans per measurement. The spectrometer compartment was continuously purged with dry air to minimize atmospheric water vapor interference. A background spectrum was recorded under identical conditions before each measurement series. Spectral acquisition and processing, including baseline correction and normalization, were performed using OPUS 7.0 software (Bruker, Ettlingen, Germany).

### 4.5. IR Microscopy Mapping

FTIR microspectroscopy and chemical mapping of polyurethane MIPs were performed using an MICRAN-3 FTIR microscope (Simex, Novosibirsk, Russia). A small amount of the dried polymer particles was deposited onto an infrared-transparent substrate and analyzed at room temperature. Initially, the sample surface was scanned using a coarse spatial step to identify representative regions of interest. Subsequently, selected areas were examined at higher spatial resolution, and spectral maps were acquired over regions measuring 10 × 10 μm.

### 4.6. Dynamic Light Scattering (DLS)

ζ-Potential measurements were performed using a Zetasizer Nano S analyzer (Malvern Instruments, Malvern, Worcestershire, UK) equipped with a 633 nm He–Ne laser at 25 °C. The samples were diluted in Milli-Q water prior to zeta potential measurements. Each sample was analyzed in triplicate, and the results were processed using Zetasizer Software v.6.32 (Malvern Instruments, UK). The obtained ζ-potential values are reported as mean ± standard deviation.

### 4.7. Nanoparticle Tracking Analysis (NTA)

NTA measurements were performed using a NanoSight LM10-HS instrument (NanoSight Ltd., Malvern, UK). The β-cyclodextrin-based polymers were dissolved in Milli-Q water (1–2 μg/mL). Each sample was measured five times, and the results are presented as mean ± standard deviation. The average molecular weight (*M_w_*) was estimated from the polymer concentration and particle concentration determined by NTA according to:

(1) $$M_{w}\;=\;\frac{C_{\mathrm{polymer}}}{N_{\mathrm{polymer}}}\;\times \;N_{a}$$

where *C_polymer_* is the concentration of polymer (g/mL), *N_a_* is Avogadro’s constant, and *N_polymer_* is the particle concentration (particles/mL) determined by NTA. The degree of polymerization ((DP)) was calculated as:

(2) $$DP\;=\;\frac{M_{w}}{M_{\mathrm{unit}}}$$

where *M_w_* is the calculated average molecular weight of the polymer and *M_unit_* is the molecular weight of its repeating unit.

### 4.8. Determination of Free Amino Groups

The content of free amino groups in chitosan and chitosan-based polymers was determined by spectrophotometric titration with TNBS. Polymer solutions (0.1 mg/mL) were mixed with 0.02 M sodium tetraborate buffer (pH 9.2), followed by addition of TNBS solution. The absorbance at 420 nm was recorded for 60 min at 22 °C. The content of free primary amino groups was determined from the initial absorbance value (A_420_) obtained by extrapolation of the kinetic curve to (t = 0). A separate TNBS blank was measured under identical conditions, and the corresponding kinetic curve was used for background correction. The amino group content of the initial chitosan was taken as 100%, and the degree of amino-group modification was calculated from the decrease in (A_420_) relative to the unmodified chitosan.

### 4.9. Atomic Force Microscopy (AFM)

The morphology of the polymer samples was examined using an NTEGRA atomic force microscope (NT-MDT, Moscow, Russia) in semi-contact mode. A drop of the aqueous polymer dispersion was deposited onto a freshly cleaved mica substrate and dried at room temperature. Topographic images were acquired at room temperature and processed using the instrument software.

### 4.10. Scanning Electron Microscopy (SEM)

SEM analysis was performed using a Carl Zeiss LEO 1450 scanning electron microscope (Carl Zeiss, Jena, Germany). Prior to imaging, the samples were immobilized on conductive carbon adhesive tape and subsequently coated with a thin gold layer (~0.05–0.1 μm) by plasma spraying to provide sufficient surface conductivity.

### 4.11. Brunauer–Emmett–Teller (BET) Analysis

The specific surface area of the samples was determined by nitrogen adsorption–desorption at 77 K using a ChemiSorb 2750 analyzer (Micromeritics, Norcross, GA, USA). Prior to analysis, the samples were ground to a particle size of approximately 0.1 mm and dried at 120 °C for 5 h. The specific surface area was calculated from the adsorption isotherm using the Brunauer–Emmett–Teller (BET) equation:

(3) $$\frac{p}{{a(p}_{0}-p)}\;=\;\frac{1}{a_{m}C}\;+\;\frac{C-1}{a_{m}C}\;\times \;\frac{p}{p_{0}}$$

where *p* is the equilibrium pressure, *p*_0_ is the saturation vapor pressure of nitrogen at 77 K, *a* is the amount of adsorbed nitrogen, *a_m_* is the monolayer adsorption capacity, and *C* is the BET constant.

### 4.12. Sorption Properties

#### 4.12.1. Sorption Capacity

The sorption capacity of the synthesized MIPs was determined as follows, 10 mg of the purified and dried polymer was placed into a 2 mL eppendorf tube, and a solution of the corresponding template molecule at a predetermined concentration and required pH value (3.0, 7.4, or 10.0) was added. The solutions with pH values of 3.0 and 10.0 were prepared by adjusting the pH of aqueous solutions with hydrochloric acid and sodium hydroxide solutions, respectively. The pH was monitored using a calibrated pH meter. The samples were incubated for 24 h under constant stirring to reach sorption equilibrium. After incubation, the polymer particles were separated from the solution by filtration, and the residual concentration of the template molecule in the filtrate was determined spectrophotometrically using the corresponding calibration curve. The equilibrium sorption capacity, *Q_e_* (mg/g), was calculated according to the following equation:

(4) $$Q_{e}\;=\;\frac{(C_{0}-C_{e})\;\times \;V}{m}$$

where *C*_0_ and *C_e_* are the initial and equilibrium concentrations of the analyte, respectively, *V* is the volume of the solution, and *m* is the mass of the polymer. Non-imprinted polymers of the same composition were analyzed under identical conditions and used as controls.

The concentrations of template molecules before and after sorption were determined by UV–Vis spectroscopy using an Ultrospec 2100 Pro spectrophotometer (Amersham Biosciences, Amersham, UK) or CD-spectroscopy using a Jasco J-815 CD Spectrometer (JASCO Corporation, Hachioji, Tokyo, Japan). Absorption spectra were recorded in quartz cuvettes with an optical path length of 10 mm and a working volume of 1 mL (Hellma Analytics, Jena, Germany). Spectra were collected in the wavelength range of 200–600 nm. The analytical wavelengths corresponding to the absorption maxima of the respective template molecules were used for quantitative analysis (LV: λ_max_ = 287 nm at pH = 7.4 and 10, λ_max_ = 293 nm at pH = 3, C_min_ = 2 × 10^−6^ M; FL: λ_max_ = 436 nm at all values of pH, C_min_ = 4 × 10^−6^ M). Calibration curves were constructed for each analyte under identical experimental conditions, and analyte concentrations were calculated from the measured absorbance values. All measurements were performed at least in triplicate, and the results are reported as mean values ± standard deviation.

#### 4.12.2. Selectivity

The selectivity of the synthesized MIPs was evaluated under conditions identical to those used for sorption capacity measurements, except that the solution contained equal concentrations of the template molecule and its structural analog. After 24 h of incubation, the residual concentrations of both compounds were determined by UV–Vis spectroscopy, and the corresponding sorption capacities were calculated. The selectivity coefficient was determined as:

(5) $$\mathrm{Selectivity}\;=\frac{Q_{e,\;\mathrm{template}}}{Q_{e,\;\mathrm{analogue}\;}}$$

where *Q_e, template_* and *Q_e, analogue_* are the equilibrium sorption capacities toward the imprinted molecule and the competing structural analog, respectively. Non-imprinted polymers were analyzed under identical conditions and used as controls.

#### 4.12.3. Sorption Cycles

Dynamic sorption experiments were performed using polymer-packed columns. Briefly, 20 mg of the purified MIP was placed into a 3 mL sorption column (the column diameter is 1 cm; no additional pressure was applied), and an analyte solution with a concentration of 5 × 10^−5^ M was passed through the sorbent. All experiments were carried out at pH 3.0. After each passage cycle, the eluate was collected, and the residual concentration of the template molecule was determined by UV–Vis spectroscopy using the corresponding calibration curve. The same solution was then repeatedly passed through the column for the required number of cycles. The extraction efficiency (*R*, %) after each cycle was calculated according to the following equation:

(6) $$R\;=\frac{C_{0}\;-\;C_{n}}{C_{0}}\;\times \;10$$

where *C*_0_ is the initial analyte concentration and *C_n_* is the residual analyte concentration in the solution after the (n)-th passage through the sorbent.

### 4.13. Release Studies

Release kinetics of template molecules from the MIPs and corresponding non-imprinted controls were studied by equilibrium dialysis at pH 10.0. Briefly, 1.5 mL of a polymer–template suspension was placed into a dialysis bag with a molecular weight cut-off of 12 kDa (Serva, Heidelberg, Germany) and immersed in 1.5 mL of buffer solution at pH 10.0. The samples were incubated at 37 °C under continuous stirring on a temperature-controlled shaker. At predetermined time points, the external solution was analyzed by UV–Vis spectroscopy to determine the amount of released template molecule.

### 4.14. Fluorescence Spectroscopy

Dissociation constants (*K_dis_*) of the template molecule complexes with the synthesized polymers were determined using fluorescence spectroscopy. Measurements were performed on a Varian Cary Eclipse spectrofluorometer (Agilent Technologies, Santa Clara, CA, USA) equipped with a thermostated cell holder maintained at 37 ± 0.1 °C. Fluorescence spectra were recorded in a 10 mm quartz cuvette using excitation and emission wavelengths corresponding to the analyte under investigation (LV: λ_ex_ = 280, λ_max_ = 460, medium mode for polymers with TDI and chitosan, low mode for polymers with HMD; FL: λ_ex_ = 470, λ_max_ = 510, low mode for all samples). For determination of the dissociation constants, a solution of the template molecule at a fixed concentration was titrated with increasing amounts of the polymer. Changes in fluorescence intensity resulting from complex formation were monitored, and the dissociation constants were calculated [65], using:

(7) $$lg\frac{F_{0}\;-\;F}{F}\;=\;lg\frac{1}{K_{\mathrm{dis}}}\;+\;nlg[\mathrm{Polymer}]$$

where *F*_0_ is the fluorescence intensity of the free fluorophore in the absence of polymer, *F* is the fluorescence intensity of the fluorophore in the presence of polymer, [*Polymer*] is the polymer concentration, *K_dis_* is the dissociation constant of the fluorophore–polymer complex, and *n* is the number of binding sites.

In addition, fluorescence polarization measurements were performed using the same instrument in fluorescence polarization mode. The degree of fluorescence polarization was determined for samples containing a constant analyte concentration and increasing polymer concentrations. Dissociation constants were also independently determined from fluorescence polarization measurements using the same equation, substituting fluorescence intensity with polarization values.

### 4.15. Sorption from Milk and Blood Plasma

Model samples of milk and blood plasma were spiked with the target analyte to a final concentration of 5 × 10^−5^ M and subjected to sample preparation prior to sorption experiments. Milk samples were centrifuged at 11,000 rpm for 10 min. Subsequently, 50 μL of 10% citric acid solution per 1 mL of sample was added, followed by incubation at 37 °C for 10 min and centrifugation at 11,000 rpm for 10 min. Protein precipitation was performed by adding 1 mL of 4 M ammonium sulfate solution per 1 mL of sample, followed by incubation for 2 min and centrifugation at 11,000 rpm for 10 min. Blood plasma samples were treated with 1 mL of 4 M ammonium sulfate solution per 1 mL of sample, incubated for 2 min, and centrifuged at 11,000 rpm for 10 min.

The resulting supernatants were repeatedly passed through columns packed with 20 mg of MIP. After each sorption cycle, the residual analyte concentration was determined by UV–Vis spectroscopy, and the extraction efficiency was calculated from the decrease in analyte concentration relative to its initial value.

## 5. Conclusions

The present study demonstrates that the molecular architecture of cyclodextrin-based MIPs plays a decisive role in determining their affinity, selectivity, and release behavior. Among the investigated materials, the HPCD–TDI system prepared at a 1:1 molar ratio exhibited the most favorable combination of properties, including the highest selectivity coefficients (27.5 for levofloxacin and 84.1 for fluorescein), strong binding affinity, and efficient extraction from complex matrices. Thus, HPCD–TDI_MIP_ (1:1) can be considered the most promising and versatile sorbent among the investigated materials for selective recognition and preconcentration of the studied analytes, whereas the Chit–HPCD–Genipin_MIP_ system is advantageous when higher sorption capacity toward levofloxacin is required. The results revealed that the nature of the crosslinker significantly influences the formation of recognition sites: the rigid aromatic structure of TDI promotes the formation of highly selective cavities through additional π–π interactions with aromatic template molecules, whereas the more flexible HMD linker provides higher sorption capacities and faster binding kinetics.

The study also demonstrated that the structure and charge distribution of the template molecule strongly affect the molecular recognition process. Fluorescein and levofloxacin exhibited comparable affinities toward monomeric HPCD; however, after polymerization (MIP formation) their behavior diverged substantially. The more exposed carboxyl group of fluorescein contributes to additional interactions within the polymer matrix, leading to enhanced affinity and selectivity, whereas levofloxacin appears to be incorporated more deeply into the cyclodextrin cavity, particularly under acidic conditions. These findings indicate that not only the size and geometry of the template molecule, but also the spatial arrangement of charged functional groups, should be considered when designing MIPs with optimized recognition properties.

The synthesized MIPs retained high extraction efficiency in complex biological and food matrices, achieving up to 97.6% recovery of fluorescein from milk and blood plasma after ten sorption cycles while maintaining excellent regenerability. Collectively, the obtained results establish cyclodextrin-based MIPs as promising materials for selective sample preparation, analyte preconcentration, and controlled drug delivery, while providing practical design principles for the development of next-generation molecularly imprinted sorbents.

## Figures and Tables

**Table 1:** Properties of polymers.

Acronym	Linker	Molar Excess of Linker	Imprinted Molecule	ζ-Potential, mV	Size, nm	*M_w_*, kDa	Polymerization Degree
LV–HPCD–HMD_MIP_ (1:1)	1,6-hexame-thylene-diisocyanate	1:1	levofloxacin	27.9 (±1.0)	134 (±6)	193 (±13)	125
LV–HPCD–HMD_MIP_ (1:3)		1:3		27.0 (±1.1)	132 (±8)	184 (±16)	98
LV–HPCD–HMD_MIP_ (1:5)		1:5		29.0 (±1.5)	124 (±7)	171 (±10)	77
LV–HPCD–HMD_MIP_ (1:10)		1:10		21.1 (±2.0)	121 (±5)	198 (±18)	65
FL–HPCD–HMD_MIP_ (1:5)		1:5	fluorescein	2.0 (±0.5)	164 (±6)	211 (±15)	95
RF–HPCD–HMD_MIP_ (1:5)		1:5	rifampicin	32.8 (±1.5)	211 (±9)	242 (±23)	109
HPCD–HMD_NIP_ (1:5)		1:5	–	14.7 (±0.8)	130 (±9)	183 (±11)	82
LV–HPCD–TDI_MIP_ (1:0.5)	toluene diisocyanate	1:0.5	levofloxacin	10.0 (±0.4)	154 (±5)	199 (±16)	136
LV–HPCD–TDI_MIP_ (1:1)		1:1		8.8 (±0.3)	145 (±7)	196 (±13)	126
LV–HPCD–TDI_MIP_ (1:3)		1:3		12.4 (±0.2)	142 (±8)	183 (±9)	96
LV–HPCD–TDI_MIP_ (1:3)		1:5		17.1 (±0.7)	145 (±6)	187 (±13)	83
FL–HPCD–TDI_MIP_ (1:1)		1:1	fluorescein	−22.0 (±2.9)	208 (±5)	231 (±20)	149
RF–HPCD–TDI_MIP_ (1:1)		1:1	rifampicin	25.2 (±1.2)	243 (±7)	209 (±18)	135
HPCD–TDI_NIP_ (1:1)		1:1	–	7.5 (±0.5)	146 (±5)	194 (±14)	125
LV–Chit–HPCD–Genipin_MIP_	Genipin	1:1	levofloxacin	−6.5 (±1.8)	302 (±23)	323 (±24)	11
FL–Chit–HPCD–Genipin_MIP_		1:1	fluorescein	−15.5 (±1.8)	314 (±19)	352 (±20)	12
Chit–HPCD–Genipin_NIP_		1:1	–	12.1 (±1.9)	290 (±34)	301 (±18)	10

**Table 2:** Values of *Q_e_* (mg/g) and selectivity coefficients (pН = 3).

Polymer	*Q_e_,* mg/g	Selectivity Towards LV vs. FL	Polymer	*Q_e_,* mg/g	Selectivity Towards FL vs. LV
LV–HPCD–HMD_MIP_ (1:5)	29.8 (±1.1)	6.5 (±0.3)	FL–HPCD–HMD_MIP_ (1:5)	47.6 (±0.8)	54.9 (±3.2)
HPCD–HMD_NIP_ (1:5)	2.9 (±0.4)	1.1 (±0.3)	HPCD–HMD_NIP_ (1:5)	3.2 (±0.3)	1.3 (±0.4)
LV–HPCD–TDI_MIP_ (1:1)	23.6 (±1.2)	27.5 (±0.8)	FL–HPCD–TDI_MIP_ (1:1)	135.5 (±2.2)	84.1 (±1.7)
HPCD–TDI_NIP_ (1:1)	2.1 (±0.4)	2.4 (±0.4)	HPCD–TDI_NIP_ (1:1)	2.5 (±0.5)	1.9 (±0.3)
LV–Chit–HPCD–Genipin_MIP_	74.8 (±2.2)	13.8 (±0.6)	FL–Chit–HPCD–Genipin_MIP_	84.7 (±1.7)	32.7 (±1.1)
Chit–HPCD–Genipin_NIP_	25.5 (±0.5)	1.2 (±0.4)	Chit–HPCD–Genipin_NIP_	13.2 (±0.7)	1.8 (±0.6)

**Table 3:** Values of *K_dis_* (М), sodium phosphate-buffered solution (рН = 7.4).

Ligand	*K_dis_*, М	
	In Complex with Levofloxacin	In Complex with Fluorescein
HPCD	9.6 (±0.4) × 10^−4^	7.5 (±0.3) × 10^−4^
LV–HPCD–HMD_MIP_ (1:1)	2.8 (±0.2) × 10^−4^	3.8 (±0.3) × 10^−2^
LV–HPCD–HMD_MIP_ (1:3)	1.8 (±0.4) × 10^−4^	3.4 (±0.3) × 10^−2^
LV–HPCD–HMD_MIP_ (1:5)	1.5 (±0.3) × 10^−4^	1.0 (±0.2) × 10^−2^
LV–HPCD–HMD_MIP_ (1:10)	3.5 (±0.3) × 10^−4^	1.1 (±0.2) × 10^−2^
FL–HPCD–HMD_MIP_ (1:5)	3.4 (±0.3) × 10^−2^	2.8 (±0.4) × 10^−5^
HPCD–HMD_NIP_ (1:5) *	6.7 (±0.3) × 10^−4^	3.6 (±0.3) × 10^−4^
LV–HPCD–TDI_MIP_ (1:0.5)	6.9 (±0.3) × 10^−5^	2.0 (±0.2) × 10^−2^
LV–HPCD–TDI_MIP_ (1:1)	1.2 (±0.3) × 10^−5^	2.6 (±0.2) × 10^−2^
LV–HPCD–TDI_MIP_ (1:3)	7.5 (±0.3) × 10^−5^	2.9 (±0.3) × 10^−2^
LV–HPCD–TDI_MIP_ (1:5)	2.3 (±0.2) × 10^−4^	3.7 (±0.4) × 10^−2^
FL–HPCD–TDI_MIP_ (1:1)	2.6 (±0.3) × 10^−1^	1.2 (±0.4) × 10^−5^
HPCD–TDI_NIP_ (1:1)	4.6 (±0.2) × 10^−4^	4.2 (±0.3) × 10^−4^
LV–Chit–HPCD–Genipin_MIP_	9.5 (±0.4) × 10^−4^	2.9 (±0.2) × 10^−1^
FL–Chit–HPCD–Genipin_MIP_	2.3 (±0.2) × 10^−2^	1.1 (±0.3) × 10^−4^
Chit–HPCD–Genipin_NIP_	4.1 (±0.3) × 10^−3^	8.3 (±0.3) × 10^−3^

* The non-imprinted polymer (NIP) without a template molecule at a molar excess of 1:5, 1:1 and 1:1 for HPCD–HMD, HPCD–TDI and Chit–HPCD–G, respectively, was used as a control.

**Table 4:** Percentage of fluorescin recovered after passing through various MIPs (added 5 × 10^−5^ M fluorescin).

Сycle Number	MIPs	Percentage of Recovered Fluorescein, %	
		Milk	Blood Plasma
5	FL–HPCD–HMD_MIP_	92.1	86.0
	FL–HPCD–TDI_MIP_	71.0	66.9
10	FL–HPCD–HMD_MIP_	97.6	93.6
	FL–HPCD–TDI_MIP_	91.9	87.9

**Table 5:** Percentage of fluorescence recovered from milk samples with different initial fluorescence concentrations after 10 cycles of passing through the MIPs.

Initial Fluorescence Concentration, M	Spectroscopic Detection Method	Percentage of Recovered Fluorescein After 10 Cycle, %	
		FL–HPCD–HMD_MIP_	FL–HPCD–TDI_MIP_
1 × 10^−4^	UV–Vis	68	65
5 × 10^−5^		98	92
5 × 10^−6^	Fluorescence	96	95
5 × 10^−7^		83	80

## Data Availability

All data generated or analyzed during this study are included in this published article.

## Supplementary Materials

The following supporting information can be downloaded at https://www.mdpi.com/article/10.3390/ijms27167502/s1.
