# Supplementary material for: Molecularly Imprinted Polymers Based on Cyclodextrin Derivatives and Chitosan for Selective Extraction of Drugs and Dyes
Source: Int J Mol Sci. 2026 Aug 21;27(16):7502. doi: 10.3390/ijms27167502 (PMC13513441; doi:10.3390/ijms27167502)
Supplement: Supplementary file 1 [file ijms-27-07502-s001.zip › ijms-4462929-supplementary.pdf]

International Journal of
Molecular Sciences

MDPI

Supplementary Materials

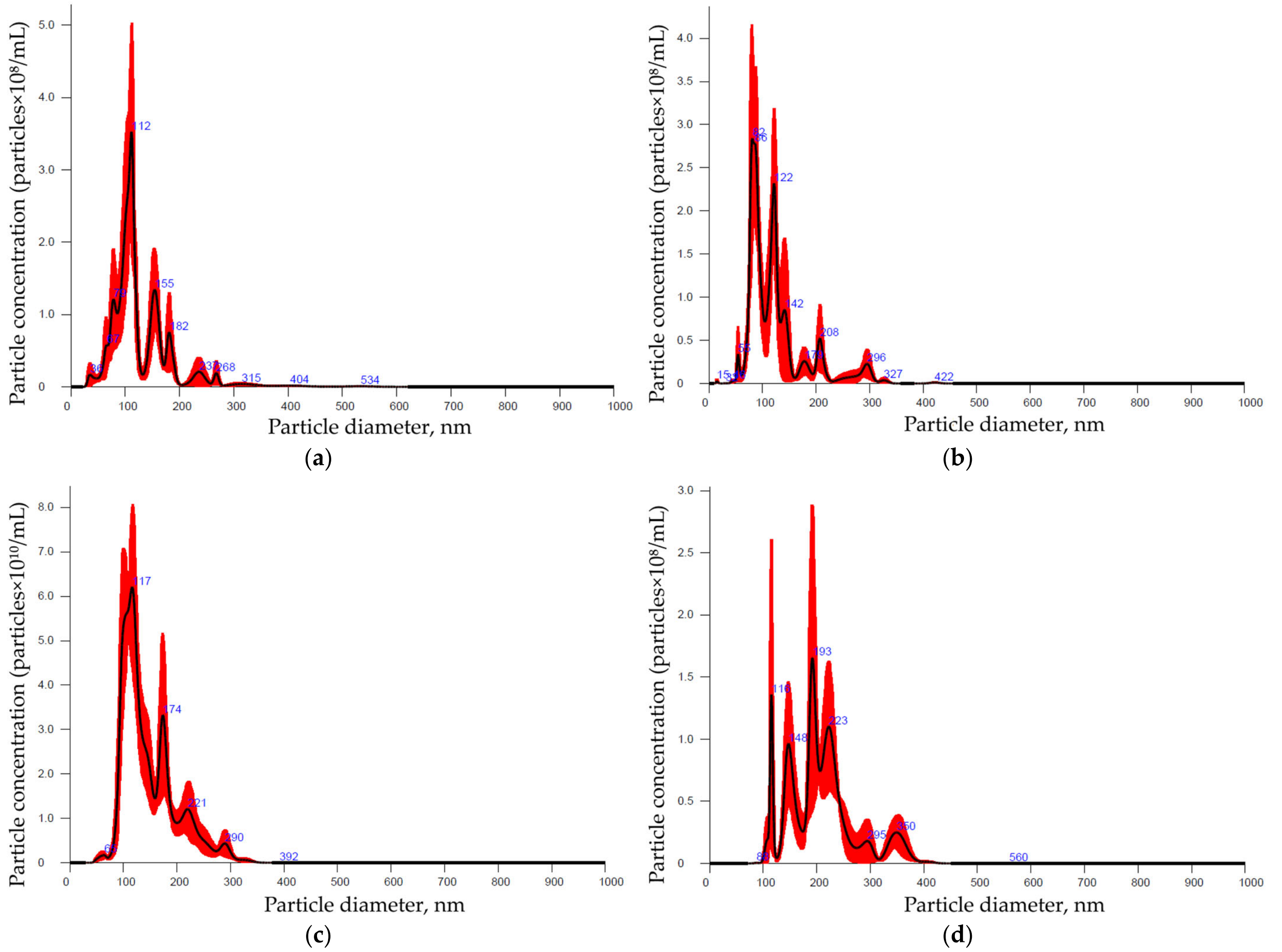

Figure S1. Particle size distribution diagram for **(a)** HPCD–HMD$_{NIP}$(1:5); **(b)** LV–HPCD–HMD$_{MIP}$(1:5); **(c)** HPCD–TDI$_{NIP}$(1:1); **(d)** FL–HPCD–TDI$_{MIP}$(1:1). The data were obtained using the NTA method.

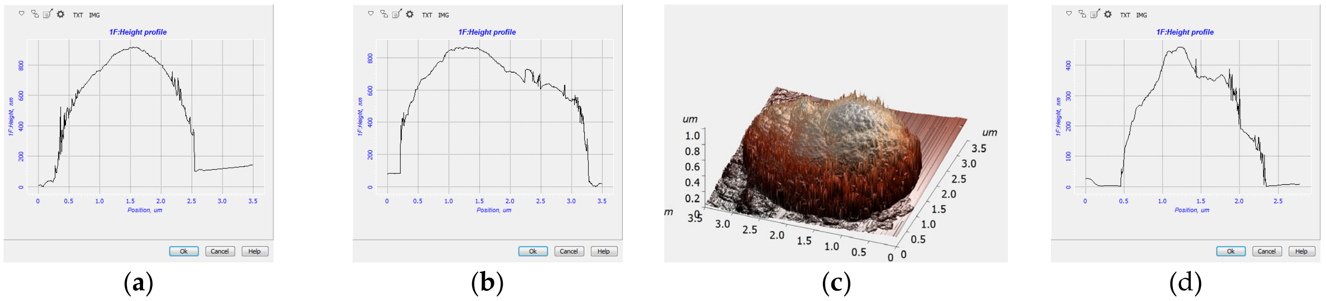

Figure S2. Particle surface profile **(a, b, c)** HPCD–HMD$_{NIP}$(1:5); **(d)** LV–HPCD–HMD$_{MIP}$(1:5). The data were obtained using the AFM method.

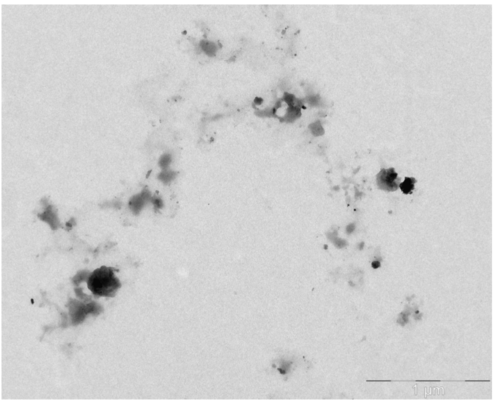

Figure S3. SEM image of the non-imprinted HPCD–HMD polymer (HPCD–HMD_{NIP}(1:5)).

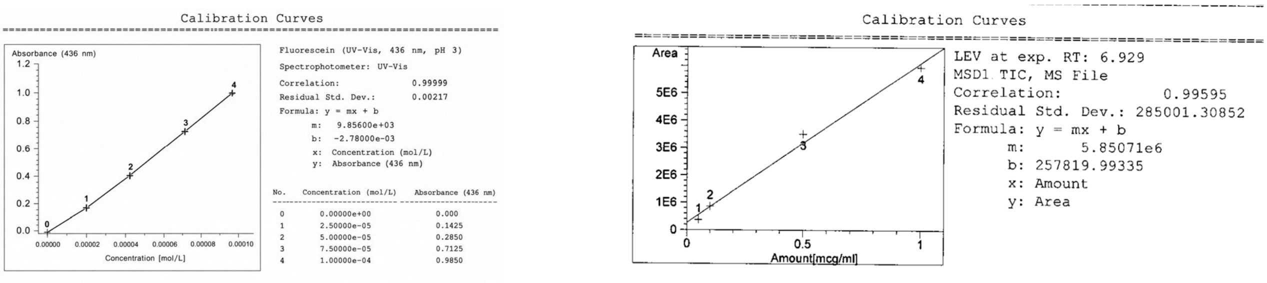

(**a**) (**b**)

Figure S4. (a) Calibration curve for the substance fluorescein in the milk; **(b)** calibration curve for the substance leaofloxacin in blood using Agilent LC/MSD ChemStation B.03.02-SR2 software.

https://doi.org/10.3390/ijms27167502
